# Lysophosphatidic acid acyltransferase 2 and 5 commonly, but differently, promote seed oil accumulation in *Brassica napus*

**DOI:** 10.1186/s13068-022-02182-2

**Published:** 2022-08-12

**Authors:** Kai Zhang, Jianjie He, Yongtai Yin, Kang Chen, Xiao Deng, Peng Yu, Huaixin Li, Weiguo Zhao, Shuxiang Yan, Maoteng Li

**Affiliations:** Department of Biotechnology, College of Life Science and Technology, Huazhong University of Science and Technology, Wuhan, 430074 China

**Keywords:** *Brassica napus*, Lysophosphatidic acid acyltransferase, Seed oil accumulation, Transcriptome, Phosphatidic acid, Triacylglycerol

## Abstract

**Background:**

Increasing seed oil content (SOC) of *Brassica napus* has become one of the main plant breeding goals over the past decades. Lysophosphatidic acid acyltransferase (LPAT) performs an important molecular function by regulating the production of phosphatidic acid (PA), a key intermediate in the synthesis of membrane and storage lipids. However, the mechanism underlying the effect of LPAT on the SOC of *B. napus* remains unclear.

**Results:**

In the present study, significant elevation of SOC was achieved by overexpressing *BnLPAT2* and *BnLPAT5* in *B. napus*. RNAi and CRISPR–Cas9 were also successfully used to knock down and knock out these two genes in *B. napus* where SOC significantly decreased. Meanwhile, we found an accumulation of lipid droplets and oil bodies in seeds of *BnLPAT2* and *BnLPAT5* overexpression lines, whereas an increase of sugar and protein in *Bnlpat2* and *Bnlpat5* mutant seeds. Sequential transcriptome analysis was further performed on the developing seeds of the *BnLPAT2* and *BnLPAT5* overexpression, knockdown, and knockout rapeseed lines. Most differentially expressed genes (DEGs) that were expressed in the middle and late stages of seed development were enriched in photosynthesis and lipid metabolism, respectively. The DEGs involved in fatty acid and lipid biosynthesis were active in the overexpression lines but were relatively inactive in the knockdown and knockout lines. Further analysis revealed that the biological pathways related to fatty acid/lipid anabolism and carbohydrate metabolism were specifically enriched in the *BnLPAT2* overexpression lines.

**Conclusions:**

*BnLPAT2* and *BnLPAT5* are essential for seed oil accumulation. *BnLPAT2* preferentially promoted diacylglycerol synthesis to increase SOC, whereas *BnLPAT5* tended to boost PA synthesis for membrane lipid generation. Taken together, *BnLPAT2* and *BnLPAT5* can jointly but differently promote seed oil accumulation in *B. napus*. This study provides new insights into the potential mechanisms governing the promotion of SOC by *BnLPAT2* and *BnLPAT5* in the seeds of *B. napus*.

**Supplementary Information:**

The online version contains supplementary material available at 10.1186/s13068-022-02182-2.

## Introduction

Plant oils are an important agricultural commodity and are composed predominantly of triacylglycerol (TAG) [1, 2]. In the developing seeds of oilseed crops, such as soybean and rapeseed, seed TAG serves as energy storage that supports seedling germination and growth. Storage lipids also have food and feed applications and provide renewable and sustainable feedstock that can be a potential alternative to petroleum in many industrial applications [3]. The demand for vegetable oils is predicted to increase by more than 40% in the next decade as a result of global population explosion and the reduction in arable land [4, 5]. Improving oil productivity is therefore an important agronomic goal of plant breeders worldwide.

In plants, the de novo biosynthesis of TAG mostly relies on the fatty acyl-CoA-dependent Kennedy pathway. The assembly of TAG begins from the stepwise acylation of glycerol 3-phosphate (G3P) with fatty acyl donors [6]. In detail, the sn-1 position of G3P and the sn-2 position of lysophosphatidic acid are acylated by glycerol-3-phosphate acyltransferase (GPAT) and 2-lysophosphatidic acid acyltransferase (LPAT), respectively. After the removal of the phosphate at the sn-3 position of phosphatidic acid (PA), diacylglycerol (DAG) is acylated by DAG acyltransferase (DGAT) to produce TAG. Alternatively, the acylation of PA and DAG into phosphatidylcholine (PC) or phosphatidylethanolamine could also be directly catalyzed [7]. Lysophosphatidylcholine has also been suggested to be a possible intermediate for PC transport into the chloroplast through acylation at its sn-2 position by lysophosphatidylcholine acyltransferase [2, 7]. Some studies have reported great achievements in increasing the oil contents of seed and non-seed tissues [8–12]. For example, in *Brassica napus*, *BnLEC1* overexpression could increase seed oil content (SOC) from approximately 47.12% to 54.73% [8]. Under normal and drought conditions, SOC was obviously increased in the *BnVOC* and *BnLEA3* overexpression lines of *B. napus* [9]. Knocking out *TRANSPARENT TESTA 8* resulted in seed yellowing along with a 3% increase in SOC [10]. Moreover, the synergistic effect of multiple genes greatly increases oil accumulation [13–16]. For example, the co-overexpression of *WRINKLED1*, *DGAT1*, and *OLEOSIN* resulted in up to 15% TAG accumulation in tobacco [15]. A considerable increase of 30%–33% in TAG accumulation was obtained against a transgenic background by silencing sugar-dependent1 (SDP1) lipase or overexpressing *AtLEC2* in tobacco leaf tissues [16]. The possibility for regulating underlying lipid fluxes appears to be unlimited.

Studying the function of *LPAT* genes can offer clues for increasing SOC because LPAT is a key enzyme that is involved in the biosynthesis of not only membrane phospholipids, but also storage lipids in developing seeds. Five *LPAT* genes, namely, *LPAT1*, *LPAT2*, *LPAT3*, *LPAT4*, and *LPAT5*, have been characterized in Arabidopsis. Except for *AtLPAT1*, which encodes plastid LPAT, others (*ATLPAT2–5*) encode cytoplasmic LPATs [17]. They have essential roles in TAG biosynthesis and plant growth and development [18–21]. Overexpression of *CrLPAT1* in the microalga *Chlamydomonas reinhardtii* could lead to a considerable increase in oil content under nitrogen-deficient conditions [22]. TAG was found to have decreased by up to 50% in the *lpat2;3*, *lpat2;4*, and *lpat3;4* double mutants of *Nannochloropsis* [23]. In oilseed crops, overexpression of *RcLPAT2* in Lesquerella increased seed tri-HFA-TAG content from 5 to 13–14% [24]. The seed-specific overexpression of *AhLPAT2* could lead to an increase of 32.2% in the oil content of Arabidopsis [25]. Medium-chain fatty acids at the sn-2 position of TAG accumulated when *CpuLPATB* and *CvLPAT2* were co-expressed in Camelina [26].

Rapeseed (*B. napus* L. AACC, 2*n* = 38) is one of the most important edible oilseed crops that is cultivated worldwide because of its considerable yield output and high nutritional value [27]. In rapeseed, oil is mainly stored in the form of TAG, which accounts for 40–60% of the dry seed weight. An increase of 7% in the SOC of rapeseed is equivalent to a yield increase of 16% in oil production [28]. Hence, increasing SOC has always been a major goal in rapeseed breeding. Previously, QTL mapping in our lab identified *BnLPAT2* and *BnLPAT5* from two individual natural populations (that is, the KN and TN populations) of *B. napus* as potential genes located in the candidate regions for SOC [29–31]. The phylogenetic relationship and synteny analyses of *LPAT* homologous genes from four related Brassicaceae species revealed four *BnLPAT2* homologous genes and four *BnLPAT5* homologous genes that are closely related to *AtLPAT2* and *AtLPAT5*, respectively [32]. The heterogeneous expression of a mutated yeast sn-2 acyltransferase increased the SOC of high-erucic acid *B. napus* [33]. Furthermore, the overexpression of *Tropaeolum majus LPAT* genes could lead to a 25–29% increase in the TAG content of seeds with altered fatty acid distributions [34]. Nevertheless, the functional analysis of these different *BnLPAT* homologous genes has been rarely reported in *B. napus*.

In the present study, we characterized the function of *BnLPAT2* and *BnLPAT5* by analyzing the SOC, seed microstructure, and gene expression patterns of the CRISPR–Cas9-mediated knockout, overexpression, and RNAi-mediated knockdown transgenic lines of *B.* *napus*. We demonstrated that *BnLPAT2* and *BnLPAT5* are essential for the increase in SOC via a distinct regulatory mechanism.

## Results

### Identification of the *BnLPAT2* and *BnLPAT5* knockout, knockdown, and overexpression lines of *B. napus*

**Fig. 1:**
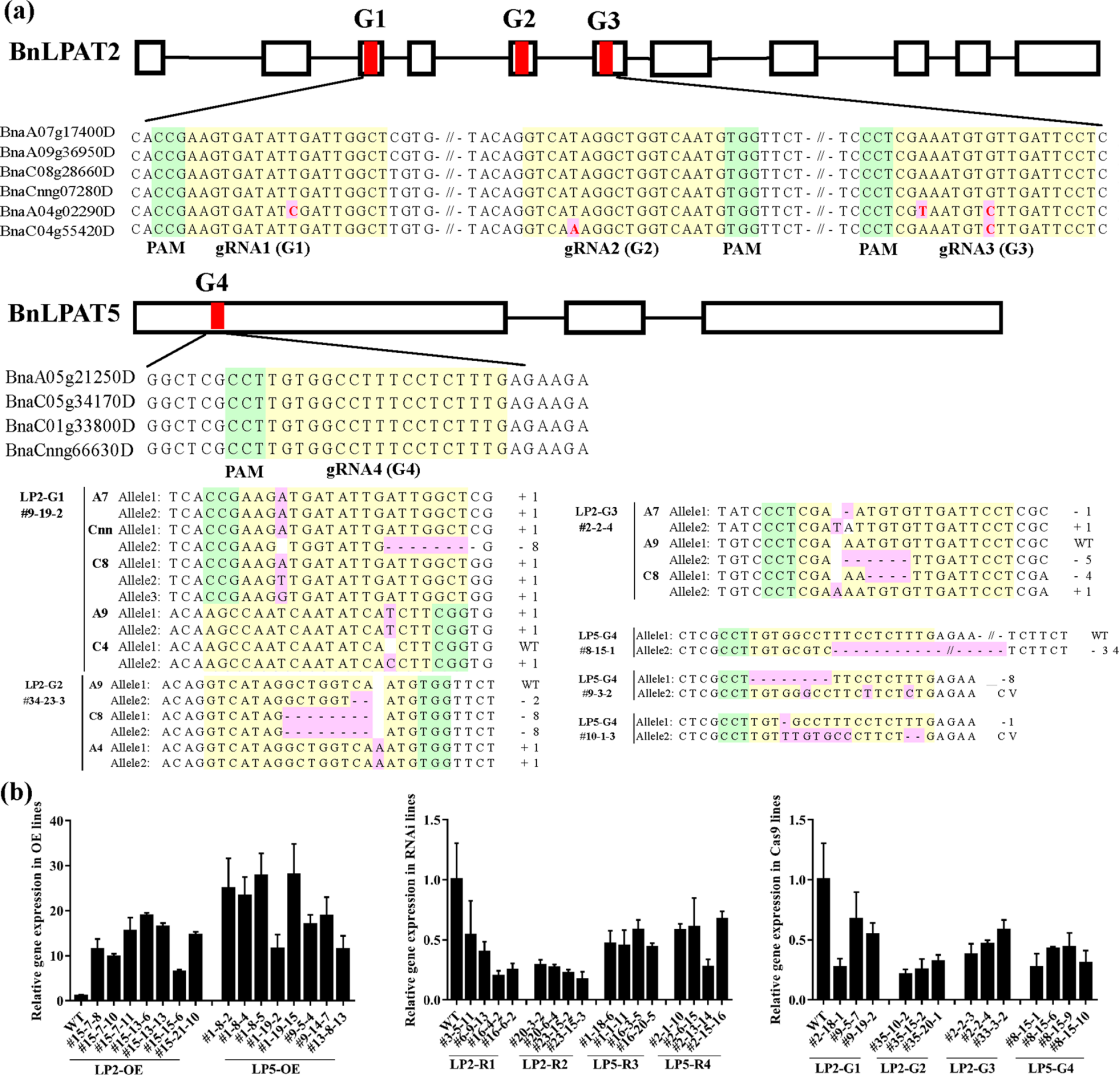
Molecular identification of different T3 *BnLPAT2* and *BnLPAT5* lines. **a** CRISPR–Cas9 induced target mutations in the T3 progenies of *BnLPAT2*&*5* knockout plants. Exons are shown as rectangles, whereas introns are shown as black lines. Target sites are located in conserved CDS regions. PAMs are in green, and gRNAs are in yellow. SNPs are highlighted in light red. “ + ” and “ − ” indicate nucleotide insertions and deletions in the target sequences, respectively; “s” indicates substitutions; “c” indicates combinatorial mutations; and “cv” indicates complicated variant. **b** Relative gene expression levels of *BnLPAT2*&*5* in the corresponding overexpression, RNAi, and Cas9 lines

Three and one gRNAs were designed for targeting *BnLPAT2* and *BnLPAT5*, respectively, to generate the CRISPR–Cas9-induced knockout mutants of *BnLPAT2*&*5*. Vectors harboring the corresponding gRNAs were then introduced into the semi-winter variety ‘J2016’ via *Agrobacterium*-mediated hypocotyl transformation, and four CRISPR–Cas9-mediated *Bnlpat2* and *Bnlpat5* knockout T1 mutant lines were obtained, i.e., LP2-G1, LP2-G2, LP2-G3, and LP5-G4 [32]. The identified T1 mutants with decreased SOCs were planted in successive years, and the T2 and T3 lines were obtained through consecutive self-crossing. Finally, 155 CRISPR–Cas9-mediated T3 mutant plants were obtained (Additional file 1: Table S1). All the homologous copies of the *BnLPAT2* gene were completely knocked out in most of the LP2-G1 lines of the T3 generation; however, only partial copies of the *BnLPAT2* gene were knocked out in most of the LP2-G2 and LP2-G3 lines (Fig. 1a). Meanwhile, most of the LP5-G4 knockout lines were chimeric at the target region (Fig. 1a). The sequencing of the T3 knockout lines revealed that the editing types consisted of insertion, deletion, substitution, and combinatorial mutations. Similar to those in T1 progenies, the lengths of the deleted sequence in T3 ranged from 1 to 34 bp, and single-base insertion and deletion almost occurred at the position of 3 bp upstream from PAM (Fig. 1a). In consideration of the continuous cleavage activity of Cas9, herein, one *Bnlpat5*-Cas9 line (T3-G4-8-15-9) was crossed with three distinct cultivars, namely, J9709, QT001, and QT002. Strikingly, effective editing in the target region of these rapeseed varieties was detected (Additional file 2: Figure S1), indicating the powerful cleavage activity of Cas9 even in the T3 generation.

In addition to the generation of knockout lines, overexpression and RNAi vectors were introduced to obtain transgenic *B. napus* [35]. The overexpression lines were named LP2-OE and LP5-OE, and the RNAi-mediated knockdown lines were designated as LP2-R1, LP2-R2, LP5-R3, and LP5-R4. After self-pollination, 384 T2 and 357 T3 transgenic plants were obtained (Additional file 1: Table S1). Further analysis demonstrated that the expression levels of the *BnLPAT2* and *BnLPAT5* genes in the LP2-OE and LP5-OE lines were 0.9–17.9 and 5.8–27.0 times higher than those in the wild type (WT), respectively (Fig. 1b). However, compared with those in the WT, the expression levels of the *BnLPAT2* and *BnLPAT5* genes in the *Bnlpat2* and *Bnlpat5* knockdown and knockout lines had greatly decreased by up to 70% (Fig. 1b).

### *BnLPAT2* and *BnLPAT5* redundantly influenced seed oil accumulation in *B. napus*

**Fig. 2:**
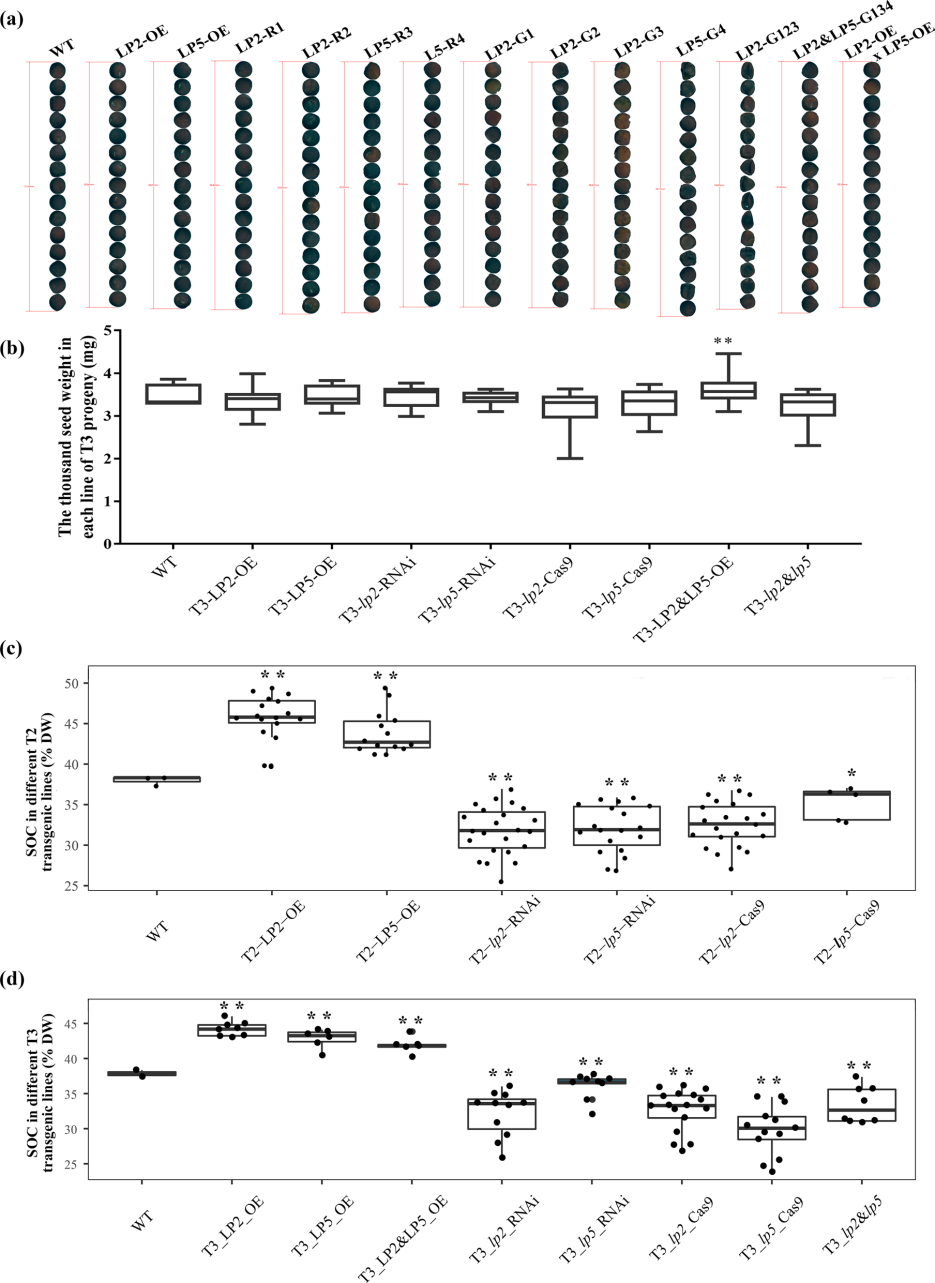
Analysis of the SOCs and morphologies of the mature seeds of different *BnLPAT2* and *BnLPAT5* transgenic lines. **a** Seed morphology of the dry mature seeds of different *BnLPAT2* and *BnLPAT5* transgenic lines. The seeds are laid out from the largest to the smallest in terms of area. **b** TSWs of different *BnLPAT2* and *BnLPAT5* transgenic lines. **c**, **d** SOCs of the T2 and T3 progenies of different *BnLPAT2* and *BnLPAT5* transgenic lines. The small black dots in **c** and **d** represent the SOCs of different biological samples. LP2-G123 and LP2&LP5-G134 are multi-gRNA-mediated knockouts of *BnLPAT2* and *BnLPAT5* from G1 to G4 as described in a previous work [32]. LP2-OE&LP5-OE represents the hybrid lines of LP2-OE and LP5-OE, and *lp2*&*lp5* represents the hybrid lines of *Bnlpat2* and *Bnlpat5* knockout or knockdown lines. Mann–Whitney test was performed on **b**, **c**, and **d**, and significant differences between transgenic lines and the WT at P < 0.05 (*) and P < 0.001 (**) are shown

Compared with the seeds of WT plants, those of the LP2-OE and LP5-OE lines were plumper and those of the *Bnlpat2* and *Bnlpat5* knockdown and knockout lines were more wrinkled and smaller; no obvious differences in thousand seed weight (TSW) were observed among these lines (Fig. 2a–b, Additional file 2: Figure S2). The SOC of WT was 37.8%. In contrast, there is a significant increase of SOC in LP2-OE and LP5-OE lines, but a decrease in *Bnlpat2* and *Bnlpat5* knockdown and knockout lines (Fig. 2c–d). Taking the T3 lines as an example, the SOCs of T3-LP2-OE and T3-LP5-OE had increased by an average of 16.9% and 13.2% to 43.0%–46.0% and 40.4%–44.2%, respectively, compared with those of the WT (Fig. 2d). By contrast, when compared with those of the WT, the SOCs of the *BnLPAT2* and *BnLPAT5* knockdown lines had decreased by 15.6% and 5.4% on average, respectively. The SOCs of the *Bnlpat2*-Cas9 and *Bnlpat5*-Cas9 lines also displayed reductions of 13.8% and 15.4% on average. These results illustrated that *BnLPAT2* and *BnLPAT5* indeed play critical roles in the enhancement of seed oil accumulation.

Crossing experiments were also performed to investigate the cooperative effect of *BnLPAT2* and *BnLPAT5* on SOC. The *BnLPAT2&5* co-overexpression lines had obviously heavier TSWs than the WT. The SOCs of the *BnLPAT2&5* co-overexpression lines had significantly increased by 10.6% on average to 40.1–43.8% but were lower than the SOCs of LP2-OE or LP5-OE lines. By contrast, the SOCs of the *Bnlpat2&5* double mutants had significantly decreased to 30.8% by 13.4% on average (Fig. 2d). These results implied that *BnLPAT2* and *BnLPAT5* have redundant functions in seed oil accumulation.

### Microstructure of *BnLPAT2* and *BnLPAT5* transgenic seeds

**Fig. 3:**
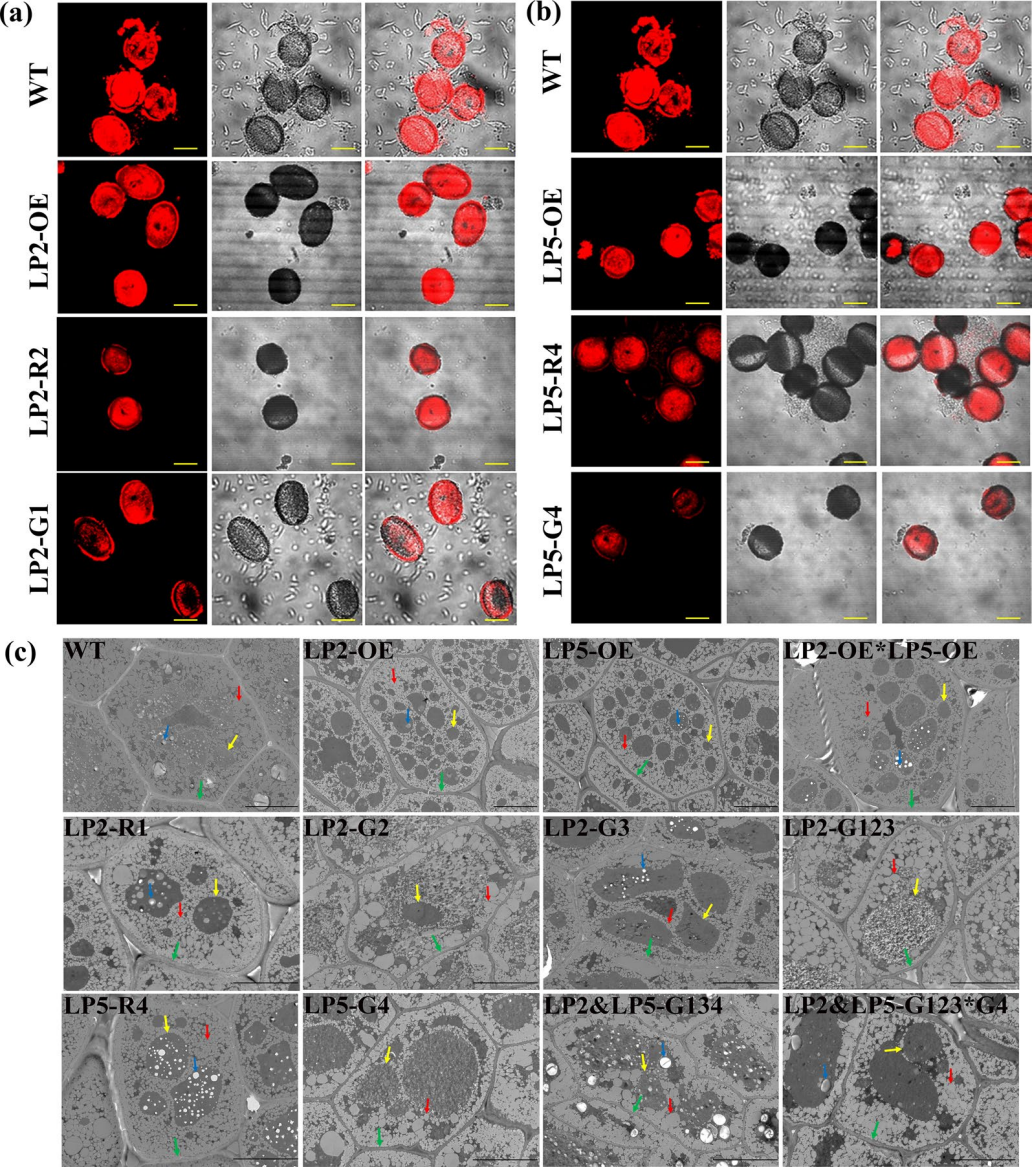
Microstructural observation of lipid droplets in *B. napus* embryos. **a** and **b** The microstructure of lipid droplets in the mature pollens of the *BnLPAT2* and *BnLPAT5* transgenic lines. Scale bar, 20 μm. **c** Microstructure of cotyledons in the mature seeds of different *BnLPAT2* and *BnLPAT5* transgenic lines. LP2-G123 and LP2&LP5-G134 are the multi-gRNA-mediated knockouts of *BnLPAT2* and *BnLPAT5* from G1 to G4 as described in a previous work [32]. LP2-OE*LP5-OE represents the crossing lines of LP2-OE and LP5-OE, and LP2&LP5-G123*G4 represents the crossing lines of LP2-G123 and LP5-G4. Green arrows indicate the cell wall. Red arrows indicate OBs. Yellow arrows indicate PBs. Blue arrows indicate starch. Scale bar, 10 μm

Lipid droplets in mature pollens are critical for seed formation. These structures were first inspected through confocal laser scanning microscopy. The intensity of the red fluorescence signal of the lipid droplets in the mature pollens of the LP2-OE and LP5-OE lines was greater than that in the WT and lower than that in the *Bnlpat2* and *Bnlpat5* knockdown and knockout lines (Fig. 3a–b).

Subsequently, the cotyledons of the mature seeds of the T2 and T3 transgenic plants were examined by using a transmission electron microscope (TEM) (Fig. 3c, Additional file 2: Figure S3). The cell sizes of the LP2-OE and LP5-OE lines had increased by 1.71- and 1.45-fold on average, respectively (Additional file 2: Figure S4a). The cell size of the *BnLPAT2&5* co-overexpression transgenic lines was 1.8 times larger than that of the WT and was also slightly larger than that of the LP2-OE or LP5-OE plants. The cell sizes of the *Bnlpat2*&*5* knockdown and knockout lines varied across lines. For example, the cell sizes of the T3 generation of *Bnlpat5*-Cas9 and *Bnlpat2*-Cas9 mutants had decreased by 14.1% and 20.1% relative to those of the WT, respectively, but increased in those of the LP2-R1, LP5-R4, and LP2&LP5-G134 lines.

Individual cells were then carefully examined. In the WT and overexpression lines, the oil bodies (OBs) were mostly uniform in size and evenly distributed in the periphery of the cells or were surrounded by protein bodies (PBs) (Fig. 3c, Additional file 2: Figure S3). The intracellular distances between the OBs in the overexpression lines were narrower than those between the OBs in the knockdown and knockout lines. The OBs in the RNAi and Cas9 lines were heterogeneous in size and fewer than those in the overexpression lines. The co-occurrence of ultra-small and large OBs was indicative of the reduction in the total occupied OB volume in a single cell and implied that the SOCs of the knockdown and knockout lines had decreased. Some differences in PB organization were observed in different lines. As illustrated in Fig. 3c, compared with the WT, the overexpression lines harbored larger amounts of PBs with smaller sizes, whereas the knockdown and knockout lines had considerably larger PBs. In addition, the ratio of PB area to cell area in the overexpression lines was very similar to that in the WT but was significantly increased in the knockdown and knockout lines (Additional file 2: Figure S4b). The organization of OBs and other subcellular compartments in the LP2-OE&LP5-OE co-overexpression lines was the same as that in the LP2-OE or LP5-OE transgenics. Nevertheless, similar to those of the OBs in LP2-G123 and LP5-G4 individuals, the amounts and sizes of the OBs in the Cas9-induced double mutant lines had decreased. The large cells of the overexpression lines could accommodate large amounts of OBs, which are positively correlated with the SOC. This finding indicated that *BnLPAT2* and *BnLPAT5* contribute to seed oil accumulation in *B. napus*.

### Different gene expression profiles in the *BnLPAT2* and *BnLPAT5* transgenic lines during seed development

RNA-seq analysis was performed on the seeds that showed differences in gene expression and SOCs at 14, 26, and 38 days after flowering (DAF) to investigate the gene expression profiles of the *BnLPAT2* and *BnLPAT5* transgenic lines during seed development (Fig. 1, Additional file 1: Table S2, Additional file 2: Figure S5). A total of 63 samples were analyzed, and more than 3.4 billion clean reads were generated (Additional file 1: Table S3). Pearson correlation coefficients between any two of the three biological replicates in the transgenic and WT lines at each stage ranged from 0.95 to 0.99 (Additional file 2: Figure S6–S8). Eleven genes were randomly selected for RT-qPCR analysis, and the expression tendency was in line with the RNA-seq results (Additional file 2: Figure S9). The number of differentially expressed genes (DEGs) at the middle and late seed development stages was higher than that at the early stage in the *BnLPAT2* and *BnLPAT5* transgenic lines, and the number of DEGs or TFs that were specifically expressed in *BnLPAT2* transgenics was considerably higher than that in the *BnLPAT5* transgenic lines at each developmental stage, except for G1 and G4 at 26 and 38 DAF (Additional file 1: Table S4–S5, Additional file 2: Figure S10–S12). These results indicated that gene expression profiles comprehensively changed during the middle to the late stages of seed development and that distinct expression patterns between *BnLPAT2* and *BnLPAT5* transgenics might underlie the different regulatory mechanisms of seed oil accumulation.

**Fig. 4:**
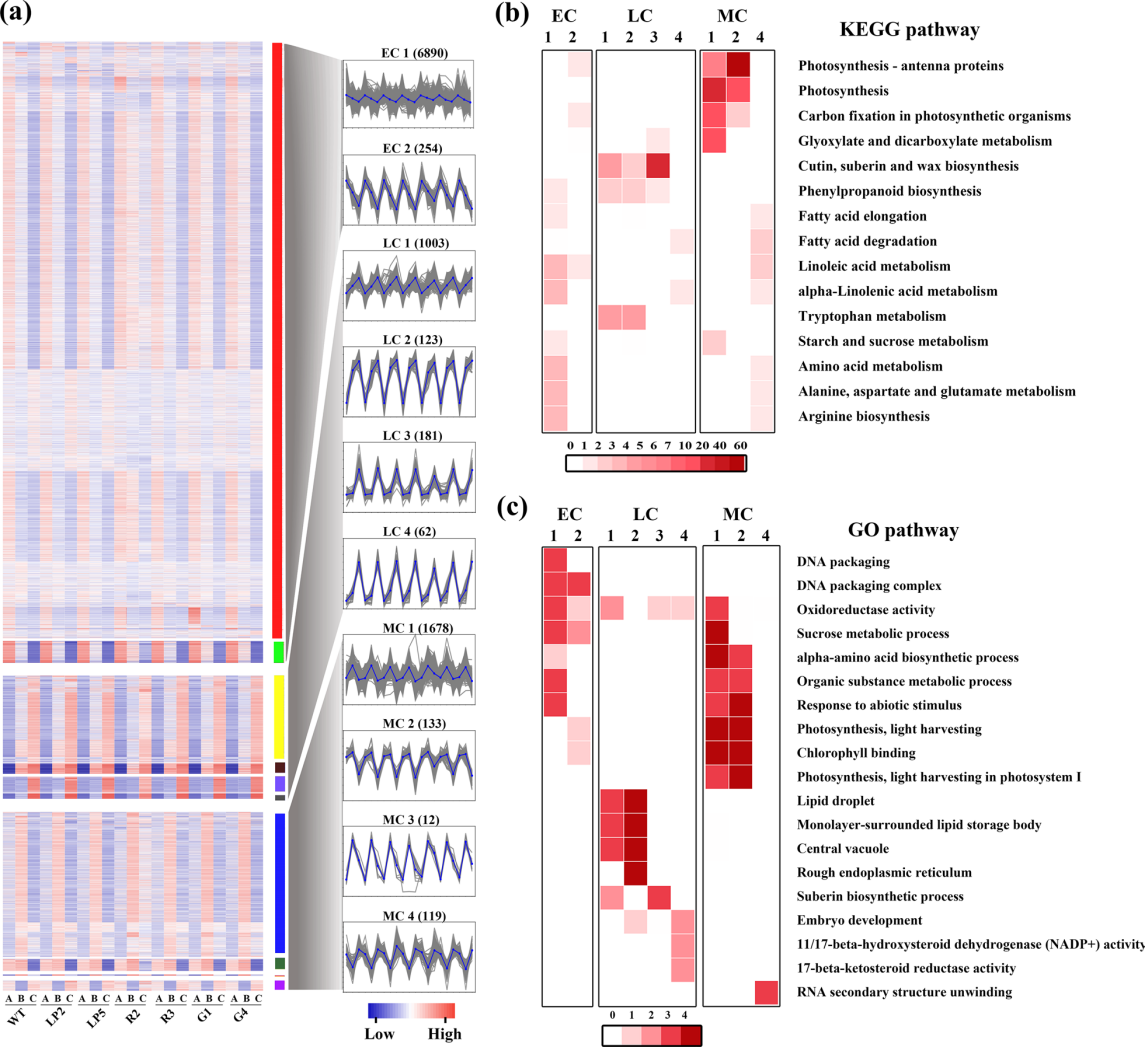
Analysis of the temporal expression patterns and relationship between the *BnLPAT2* and *BnLPAT5* transgenic lines. **a** Temporal expression patterns of all of the DEGs in the *BnLPAT2* and *BnLPAT5* lines. Gray shaded markings represent the corresponding relationship between the left and right panels. **A**–**C** Represent samples at 14, 26, and 38 DAF. **b** KEGG pathway analysis of DEGs in the *BnLPAT2* and *BnLPAT5* lines. **c** GO pathway analysis of DEGs in the *BnLPAT2* and *BnLPAT5* lines

The DEGs were classified into three groups: (1) early cluster (EC), which included 7144 DEGs and two subclusters and showed the highest expression levels only at the early stage; (2) late cluster (LC), which included 1369 DEGs and four subclusters and exhibited the highest expression levels only at the late stage; and (3) middle cluster (MC), which included 1942 DEGs and four subclusters and presented the highest or lowest expression levels at the middle stage (Fig. 4a). The DEGs in MC1 and MC2 were significantly enriched in photosynthesis, photosynthesis-antenna proteins, carbon fixation in photosynthetic organisms, and glyoxylate and dicarboxylate metabolism. These pathways are associated with energy and carbohydrate metabolism. However, the expression levels of these genes did not significantly differ among different transgenic lines (Fig. 4, Additional file 1: Table S6, Additional file 2: Figure S13), suggesting that photosynthesis might be a greatly important, but not decisive, factor for seed oil accumulation at 26 DAF. In addition, the DEGs in LC3 were significantly enriched in cutin, suberin and wax biosynthesis and were up-regulated in the R2/R3 and G1/G4 lines but were down-regulated in the LP2/LP5 lines relative to those in the WT (Fig. 4, Additional file 1: Table S6, Additional file 2: Figure S13). These results confirmed that lipid metabolism is a key factor for seed oil accumulation at 38 DAF. The enrichment of the DEGs in LC1–3 in the metabolic pathways of other amino acids, such as tryptophan metabolism, and the biosynthesis of other secondary metabolites, such as phenylpropanoid biosynthesis, indicated that they have important roles in primary and secondary metabolism. Furthermore, the DEGs in ECs and MCs were mostly related to photosynthesis, sucrose metabolic process, organic substance metabolic process, and amino acid biosynthetic process that are responsible for embryo architecture and development, whereas the DEGs in LCs were mainly associated with lipid formation and accumulation (Fig. 4c). More GO terms and KEGG pathways were enriched in MCs and LCs than in ECs. These results showed that the effects of *BnLPAT2* and *BnLPAT5* on SOC at the middle and late seed development stages might be a consequence of multiple biological processes.

### Comparative analysis of seed oil accumulation in the *BnLPAT2&5* transgenic lines

**Fig. 5:**
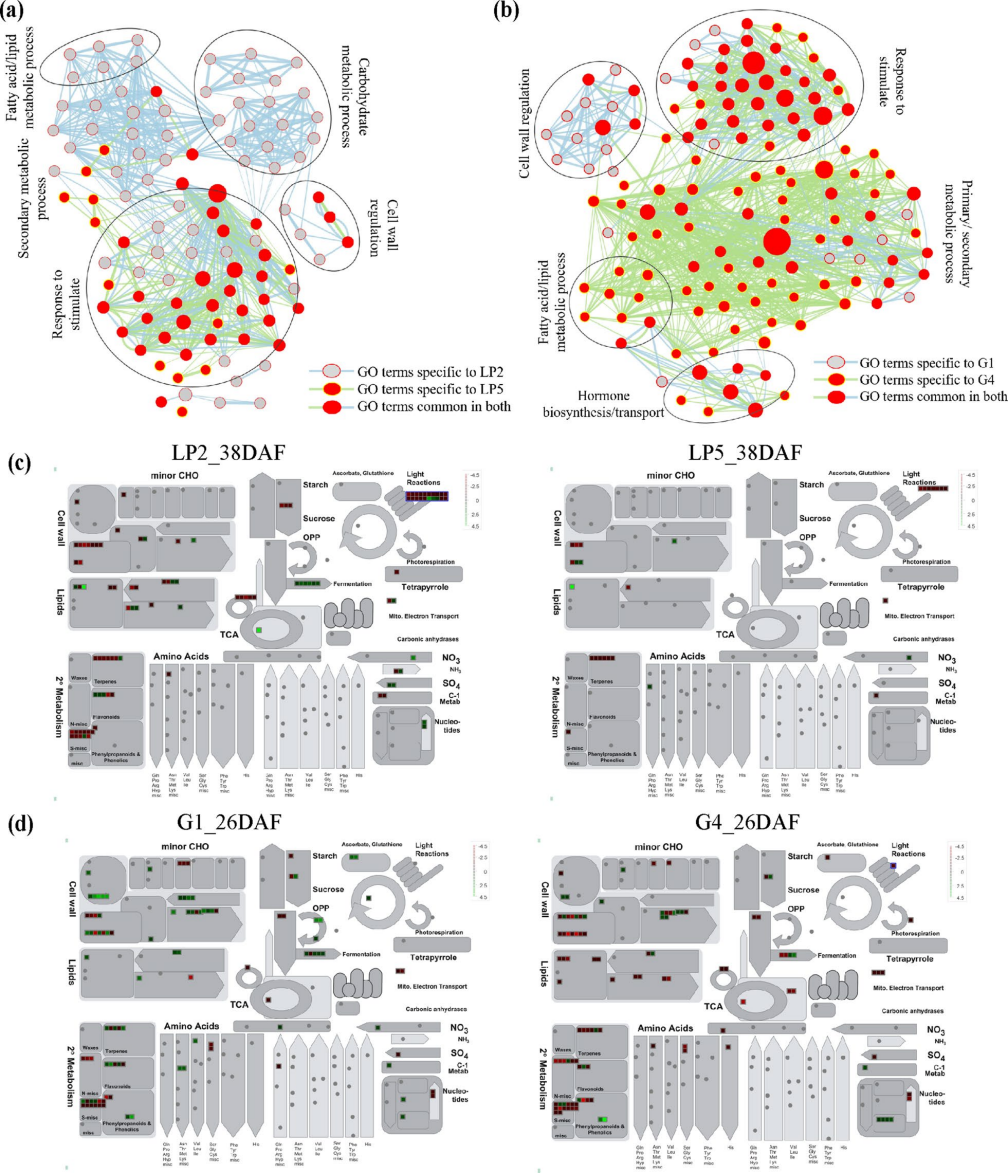
Differentially enriched pathways between the transgenic *BnLPAT2* and *BnLPAT5* lines at different stages of *B. napus* seed development. **a**, **b** GO enrichment map of DEGs in the *BnLPAT2* and *BnLPAT5* transgenic lines at all stages of seed development. The detailed GO enrichment pathways are presented in Additional file 2: Table S6, and an enlarged version of **a** and **b** with labeled GO terms is provided in Additional file 2: Figure S14. **c** and **d** MapMan metabolic pathways of the DEGs in the *BnLPAT2* transgenic lines at 26 DAF and the *BnLPAT5* transgenic lines at 38 DAF. Green and red colors represent higher and lower expression levels compared with the expression levels in the WT on the log2 scale

Overlapped GO analysis was performed to compare the differences between the *BnLPAT2* and *BnLPAT5* transgenic lines to understand oil biosynthesis and accumulation further. Secondary metabolic process, cell wall regulation, and stress-related response were the terms that were commonly enriched in LP2/LP5 and G1/G4 (Fig. 5a–b). Carbohydrate metabolic process and fatty acid/lipid metabolic process were specific to LP2 (Fig. 5a), in which lipid transfer proteins (LTPs), including LTP1, LTP5, and LTP6, were the genes that were frequently enriched (Additional file 1: Table S6). Fatty acid/lipid metabolic process was specific to G4 (Fig. 5b). Most genes involved in this process were related to cutin and suberin assembly, such as GPATs (*GPAT4* and *GPAT6*), and to the biosynthesis of very long-chain fatty acids, such as ketoacyl-coA synthase, long-chain acyl-coA synthetase 1, and long-chain acyl-coA synthetase 2 (Additional file 1: Table S6). Thus, these results indicated that *BnLPAT2* and *BnLPAT5* have different functions. Intriguingly, more pathways related to secondary metabolic process and hormone biosynthesis were specific to G1 and G4 than to LP2 and LP5, suggesting that these secondary metabolites might participate in regulating seed oil accumulation.

Then, detailed GO enrichment analysis revealed that the genes with high expression levels during seed development were significantly enriched in cell wall-related terms, carbohydrate metabolic-related process, and stress-related responses (Additional file 2: Figure S15). DEGs related to fatty acid and lipid metabolism were highly enriched in all *BnLPAT2* transgenic lines particularly at 38 DAF. Taking the TFs involved in lipid biosynthesis as an example, *WRI1* and *LEC1* showed significant up-regulation in LP2&LP5 from the early to the middle stages, whereas *ABI3* exhibited relatively high expression levels in LP2&LP5 from the middle to the late stages (Additional file 2: Figure S16). Furthermore, the DEGs in R2/R3 and G1/G4 were significantly enriched in long-chain fatty acid metabolic process and were more active than those in LP2/LP5 at 38 DAF. Long-chain fatty acids are the precursors of many secondary metabolites, such as cutin, suberin and wax. Therefore, these DEGs may actively participate in secondary metabolite biosynthesis in R2/R3 and G1/G4 at the late seed development stage. For example, *LUP2*, *TT4*, and *PDS1*, which encode the components in secondary metabolism pathways, were highly expressed in R2/R3 and G1/G4 compared with in LP2/LP5 (Additional file 2: Figure S16). The GO terms related to gibberellin metabolism were also highly enriched in R2/R3 and G1/G4. *GASA10*, the gene responsible for gibberellin synthesis, was significantly up-regulated in R2/R3 and G1/G4 (Additional file 2: Figure S16), pointing to the importance of gibberellin regulation for SOC.

MapMan analysis was also conducted, and significant differences were found in carbohydrate metabolism, oil metabolism, and secondary metabolism among the different transgenic lines (Fig. 5c–d, Additional file 2: Figure S17). During the rapid oil accumulation period (26 DAF), the DEGs involved in lipid metabolism and in the minor CHO metabolism pathway were significantly up-regulated in LP2/LP5 relative to in G1/G4. However, the DEGs involved in lipid metabolism showed higher expression levels in G1/G4 than in LP2/LP5 at 38 DAF. Further analysis revealed that at 38 DAF, the DEGs were mainly related to FA chain synthesis, FA chain extension, long-chain FA synthesis, and FA degradation. This finding indicated that lipid degradation might play a dominant role in the knockdown and knockout lines during seed maturation. In addition, substantial differences in transcriptional activity were observed under biotic stress, especially at 38 DAF (Additional file 2: Figure S17). Consistent with the GO and KEGG analysis results, the genes involved in secondary metabolites, hormone signaling, and abiotic stress were highly active in G1/G4. Collectively, these results indicated that lipid metabolism was dominant in LP2 and LP5 at 26 DAF, whereas biotic stress progress dominated in G1 and G4 at 38 DAF.

### Sugar and protein contents increased in the *BnLPAT2&5* knockdown and knockout lines

**Fig. 6:**
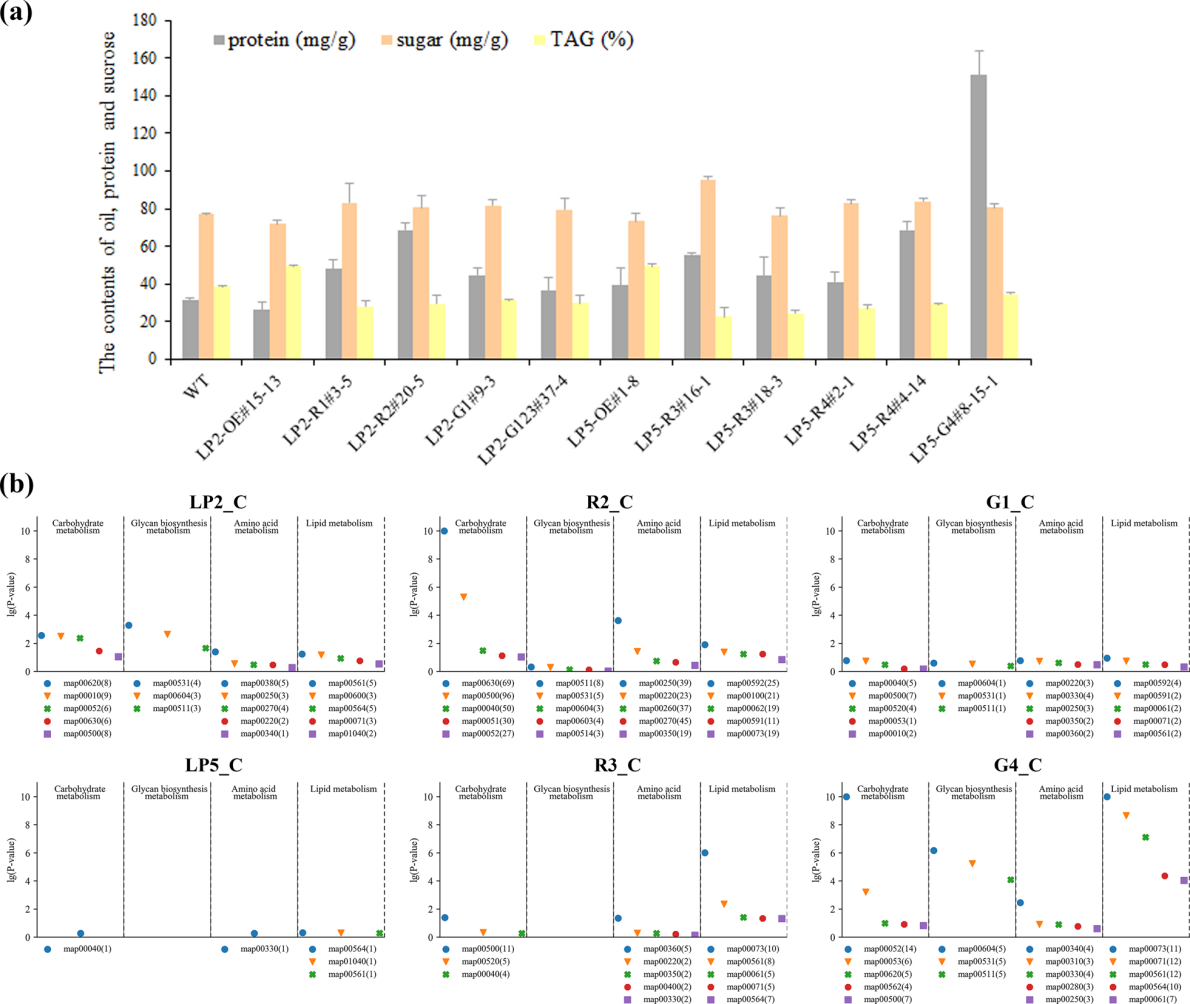
Analysis of lipid, protein, and sugar contents and metabolism in the *BnLPAT2* and *BnLPAT5* transgenic lines. **a** Total oil, protein, and sugar contents in the mature seeds of the *BnLPAT2* and *BnLPAT5* transgenic lines. LP2-G123 is the multi-gRNA-mediated knockout of *BnLPAT2* with G1, G2, and G3 as described in a previous work [32]. **b** Enriched KEGG pathways related to lipid, protein, and sugar metabolism at 38 DAF of seed development. The top five enriched KEGG pathways of major CHO metabolism, including carbohydrate metabolism, glycan biosynthesis and metabolism, amino acid metabolism, and lipid metabolism, in the *BnLPAT2* and *BnLPAT5* transgenic lines are presented. The numbers in parentheses represent the numbers of DEGs in each pathway

Dry seeds with extremely high and low SOCs were subjected to sugar and protein content analyses to investigate whether the alteration in SOCs would affect the accumulation of other seed components. The total sugar content in the WT was 77.3 mg/g, which decreased to 72.0 and 73.4 mg/g in LP2-OE and LP5-OE, respectively (Fig. 6a). However, the total sugar content of the knockdown and knockout lines increased. Specifically, the total sugar contents of the *Bnlpat2-*RNAi and *-*Cas9 plants increased to 81.8 and 80.5 mg/g, respectively, and those of the *Bnlpat5*-RNAi and -Cas9 lines increased to 84.5 and 80.7 mg/g, respectively (Fig. 6a). The total protein content showed similar alterations as sugar content, with most lines, except for LP5-G4, showing a smaller scope of change than the WT (Fig. 6a).

Then, the top five pathways were presented to further elucidate the relationships among sucrose, protein, and lipid metabolism. These pathways were significantly enriched in the *BnLPAT2* line and were higher than those in the *BnLPAT5* lines, indicating that seed oil biosynthesis might be dominantly contributed by *BnLPAT2* instead of *BnLPAT5* (Fig. 6b). Glycan biosynthesis and metabolism were more active in LP2 than in R2 and G1 of the *BnLPAT2* transgenic lines and were active in G4 but were inactive in LP5 and R3 of the *BnLPAT5* transgenic lines (Fig. 6b). Further analysis revealed that the pathways in LP2 were related to glycosaminoglycan degradation and other glycan degradation that were all closely related to glycan degradation. Glycosphingolipid biosynthesis, which is closely related to sugar biosynthesis, was identified in G4 (Additional file 1: Table S8). The increase in sugar content in the *Bnlpat2&5* knockdown and knockout lines and the decrease in sugar content in the overexpression lines may be ascribed to the above result. The DEGs involved in carbohydrate metabolism were more highly active in R2/R3 and G1/G4 than in LP2/LP5. Additionally, pyruvate metabolism and glycolysis/gluconeogenesis were mainly enriched in LP2/LP5, suggesting that the intermediate metabolites available for lipid biosynthesis, such as ATP, NADH, and acetyl-CoA, had increased. By contrast, glyoxylate/dicarboxylate metabolism and starch/sucrose metabolism were detected in R2 and G4, implying that more carbon metabolism pathways were involved in sugar storage in these lines than in the overexpression lines. The DEGs involved in lipid metabolism, such as *ATLOX2* (*BnaC06g18870D* and *BnaA07g19600D*) and *ATDOX1* (*BnaC05g46590D*), that are closely related to FA degradation were significantly enriched in R2/R3 and G1/G4 compared with those in LP2/LP5 (Fig. 6b). The enriched pathways were mainly related to alpha-linolenic acid metabolism and linoleic acid metabolism. Moreover, enriched pathways in R3 and G4 were mostly related to the production of secondary metabolites, such as cutin, suberin and wax biosynthesis and fatty acid degradation (Additional file 1: Table S8). This situation might lead to the increase in the SOC of the *BnLPAT2&5* overexpression plants and the decrease in SOC in the knockdown and knockout plants.

In addition, the considerably higher numbers of amino acid metabolism pathways that were significantly enriched in R2/R3 and G1/G4 than in LP2/LP5 indicated that protein biosynthesis and metabolism were more active in the *Bnlpat2&5* knockdown and knockout lines than in the overexpression lines. For example, *ASP1* and *ADT1* genes, which are involved in amino acid metabolism, were slightly up-regulated in the knockdown and knockout lines. These results were consistent with the GO enrichment and TEM analysis results, which also implied a negative relationship between SOC and protein content.

### *BnLPAT2* and *BnLPAT5* jointly but differently regulated seed oil accumulation in *B. napus*

**Fig. 7:**
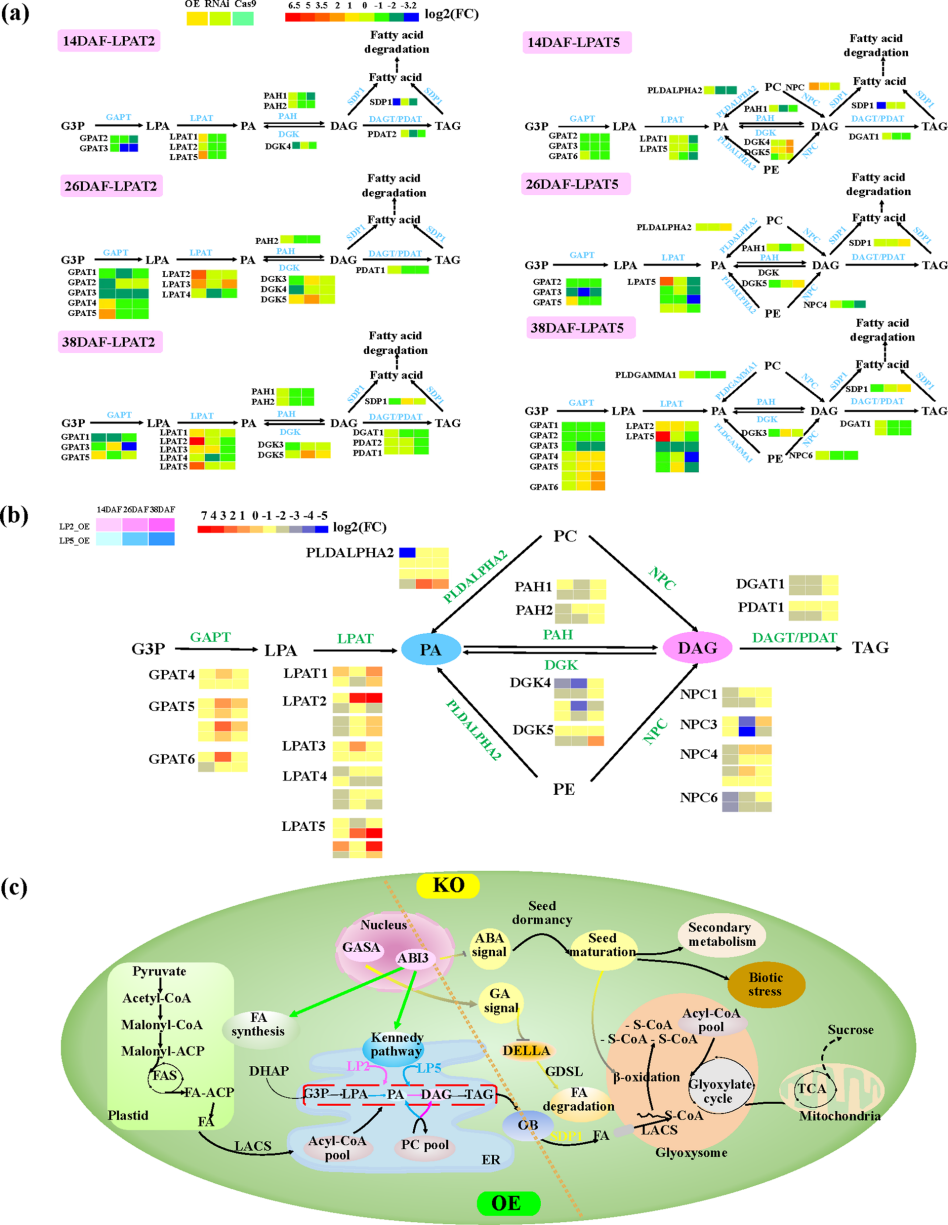
Genes involved in the TAG metabolic pathway affected by *BnLPAT2* and *BnLPAT5* in rapeseed. **a** Differential gene expression involved in TAG biosynthesis in the overexpression, knockdown, and knockout lines of *BnLPAT2* and *BnLPAT5* at three developmental stages. The expression pattern of each DEG is shown by three grids, with the first, second, and third grids from left to right representing the relative log2 (FC) of the overexpression, RNAi, and Cas9 lines, respectively. Up-regulated genes are in red, and down-regulated genes are in blue. The color indicates the value of log2 (FC). **b** Differential gene expression involved in TAG biosynthesis in LP2 and LP5 at three different seed developmental stages. The expression pattern of each DEG is shown by three grids within two rows, with the first, second, and third grids from left to right representing the relative log2 (FC) at 14, 26, and 38 DAF, respectively, and the two rows from the top to the bottom representing the relative log2 (FC) in LP2 and LP5 lines. Glycerolipid classes showing increases in LP5 lines are highlighted with a blue background and those showing increases in LP2 lines are highlighted with a pink background. **c** Proposed model of oil accumulation in *B. napus* under regulation by *BnLPAT2*&*5*. The left part of the double dotted line represents the pathways that are mainly involved in the overexpression lines, whereas the right part represents the pathways that are mainly involved in the knockdown and knockout lines. Green lines represent the enhanced pathways in LP2 and LP5. Yellow lines represent the enhanced pathways in the knockdown and knockout lines. Pink lines represent the enhanced pathways in all *BnLPAT2* transgenic lines. Blue lines represent the enhanced pathways in all *BnLPAT5* transgenic lines. *G3P* glycerol‑3‑phosphate, *LPA* lysophosphatidic acid, *PA* phosphatidic acid, *DAG* diacylglycerol, *TAG* triacylglycerol, *PC* phosphatidylcholine, *PE* phosphatidylethanolamine, *GPAT* glycerol‑3‑phosphate acyltransferase, *LPAT* lysophosphatidate acyltransferase, *DGAT* diacylglycerol acyltransferase, *PDAT* diacylglycerol acyltransferase, *DGK* diacylglycerol kinase, *NPC* nonspecific phospholipase C, *LACS* long‑chain *acyl*‑*CoA* synthetase, *DHAP* dihydroxyacetone phosphate, *OB* oil body, *GASA* Gibberellic acid stimulated Arabidopsis, *ABA* abscisic acid, *SDP1* sugar-dependent 1, *FC* fold-change, *KO* knockout and knockdown lines, OE overexpression lines

The DEGs involved in TAG biosynthesis in the *BnLPAT2* and *BnLPAT5* transgenic lines were summarized for the comprehensive characterization of the pathways of seed oil accumulation during different developmental stages (Fig. 7a). Almost all of the *LPAT* family genes were significantly up-regulated in the overexpression lines, but were down-regulated in the knockdown and knockout lines during seed development, especially at 38 DAF. From PA to DAG, the transcript levels of phosphatidic acid phosphohydrolase 1/2, which regulates the conversion of PA into DAG, were slightly up-regulated in the overexpression lines and slightly down-regulated in the knockdown and knockout lines. Similarly, genes encoding diacylglycerol kinase, which converts DAG into PA, were significantly up-regulated in the knockdown and knockout lines and significantly down-regulated in the overexpression lines at 38 DAF. In addition, the genes involved in PC/PE and PA/DAG conversion showed the opposite trends in the *BnLPAT5* transgenic lines. PLDGAMMA1 and PLDALPHA2 could promote the conversion of PC/PE into PA in the overexpression lines, but significantly inhibited the conversion of PC/PE into PA in the knockdown and knockout lines at 38 DAF. Similarly, nonspecific phospholipase C 6 also promoted the conversion of PC/PE into DAG in the overexpression lines while inhibiting PC/PE conversion in the knockdown and knockout lines at 38 DAF. These results indicated that *BnLPAT2* and *BnLPAT5* could promote DAG synthesis, which is further conducive to directing carbon sources toward TAG synthesis. Additionally, the genes encoding DGAT and PGAT, which are responsible for converting DAG into TAG, were slightly up-regulated in the overexpression lines and slightly down-regulated in the knockdown and knockout lines. Moreover, *SDP1*, which controls the degradation of DAG/TAG, was significantly up-regulated in the knockdown and knockout lines at 38 DAF, indicating that the degradation of TAG was accelerated in these two lines. Taken together, most of the up-regulated genes of the Kennedy pathway showed higher expression levels in LP2/LP5 than in R2/R3 and G1/G4, whereas the expression levels of most of the down-regulated genes were considerably lower in R2/R3 and G1/G4 than in LP2/LP5. These results suggested that the gain-of-function of *BnLPAT2*/5 could promote oil synthesis, whereas the loss-of-function of *BnLPAT2*/5 would block oil synthesis and accelerate oil degradation. In short, *BnLPAT2* and *BnLPAT5* can significantly promote seed oil biosynthesis during seed development.

The TAG biosynthesis pathways in LP2 and LP5 were analyzed in detail considering that the contribution of *BnLPAT2* to SOC was dominant over that of *BnLPAT5* (Fig. 7b). Most genes in the Kennedy pathway were significantly up-regulated in LP2 and LP5. Remarkably, the *BnLPAT2* gene resulted in a significant up-regulation of the *BnLPAT* gene family (including *BnLPAT5*), whereas *BnLPAT5* only led to the up-regulation of the homologous genes of the *BnLPAT5* family likely due to the ubiquitous expression of *BnLPAT2* in *B. napus.* In contrast to *BnLPAT5*, *BnLPAT2* plays an indispensable role throughout the whole process of plant growth and development. Therefore, the up-regulated genes in the TAG synthesis pathway were more active in LP2 than in LP5 during seed development. This phenomenon again demonstrated that the regulatory network driven by the *BnLPAT2* gene is more active and complex than that influenced by the *BnLPAT5* gene. The conversion from PC/PE into PA and DAG also differed between LP2 and LP5 (Fig. 7b). In LP2, the genes involved in the conversion from PC/PE into DAG were highly active, whereas the transformation from PC/PE into PA was suppressed especially at 14 DAF. In LP5, the conversion from PC/PE into PA was significantly enhanced, whereas the transformation into DAG weakened at 26 and 38 DAF. These results illustrated that *BnLPAT2* tends to promote the conversion from PC/PE into DAG, whereas *BnLPAT5* tends to enhance the transformation from PC/PE into PA. In addition, for the reciprocal conversion between PA and DAG in LP2 and LP5, the transformation of PA into DAG was obviously enhanced in LP2, whereas the opposite direction of transformation was dominant in LP5. Consequently, *BnLPAT2* was inferred to be capable of significantly promoting DAG synthesis, thus accelerating TAG formation in seeds. However, *BnLPAT5* might significantly promote PA synthesis and thus have a potential effect on membrane lipids.

A potential model for seed oil accumulation in *B. napus* under regulation by *BnLPAT2* and *BnLPAT5* was proposed (Fig. 7c). In this model, the expression levels of genes in the Kennedy pathway were significantly up-regulated in LP2 and LP5, and DAG and PA biosyntheses were dominant in LP2 and LP5, respectively. TAG degradation was dominant in the knockdown and knockout lines because catabolism also plays an essential role in seed oil accumulation. SDP1, which is responsible for initiating oil degradation in seeds following germination, was significantly up-regulated in the *Bnlpat2*&*5* knockdown and knockout lines. Additionally, the knockdown and knockout transgenics entered the maturity stage earlier than the overexpression lines. Therefore, gibberellic acid stimulated Arabidopsis (GASA10) and ABI3 were hypothesized to play important roles in the control of SOC by *BnLPAT2* and *BnLPAT5* (Fig. 7c). *GASA10*, which encodes the gibberellic acid-stimulated-like protein, mainly regulated seed development at 26 DAF. *GASA10* was significantly up-regulated to stimulate the GA signaling pathway in *Bnlpat2*&*5* knockdown and knockout lines and then reduced the abundance of DELLA protein to inhibit the expression of the *GDSL* gene, thereby accelerating lipid degradation. The *ABI3* transcription factor, which mainly regulates seed development at 38 DAF, was highly expressed in *BnLPAT2*/*5* overexpression lines. In the overexpression lines, *ABI3* acted as a TF to bind the RY domain of the target genes related to lipid synthesis to promote fatty acid synthesis. In the seeds of the knockdown and knockout lines, it could inhibit the accumulation of endogenous abscisic acid (ABA). This effect increased ABA content, thereby promoting the early maturation of seeds and the synthesis of secondary metabolites and antistress-related substances.

## Discussion

*LPAT* genes, which play an essential role in cellular function by controlling the biosynthesis of PA, are of great importance for membrane phospholipids and storage lipids in seeds. However, the molecular mechanisms underlying oil accumulation under the control of *LPAT* genes during seed development in *B. napus* are poorly understood. In the present study, RNA-seq was performed to investigate the molecular mechanisms underlying oil accumulation in the *BnLPAT2* and *BnLPAT5* overexpression, knockdown, and knockout lines at three different seed development stages.

### *BnLPAT2 *and *BnLPAT5* are important for oil accumulation in *B. napus*

Previously, some studies focused on a few genes involved in the TAG pathway that contributes to the SOC of rapeseed and other species. For example, the overexpression of *BnWIN1*, which leads to the up-regulation of *BnLPAT5*, contributes to an 8% increase in the SOC of *B. napus* [36]. The overexpression of *BnGPAT9* contributes to a significant increase of 6.4% in SOC [36]. The heterologous expression of *TmDGAT1* in canola leads to an increase in SOC of up to 8% [37]. In this study, we found that the overexpression of *BnLPAT2* and *BnLPAT5* led to an increase of 38.9%–49.4% in SOC. In addition, SOC decreased to 22.5% and 27.1% in the *Bnlpat2* knockdown and knockout lines and significantly decreased to 22.7% and 27.1% in the *Bnlpat5* knockdown and knockout lines (Fig. 2c–d). Moreover, an analysis of the microstructure of mature seeds showed that the cell size of the overexpression lines was larger than that of the WT, knockdown, and knockout lines. By contrast, the size and amount of PBs in the knockdown and knockout transgenics were larger than those in the overexpression lines (Fig. 3c, Additional file 2: Figure S2–S3). In addition, the reduction in the SOC of the RNAi and Cas9 lines was accompanied by the increase in the accumulation of total protein and sugar contents (Fig. 6a). The negative correlation between oil and protein/sugar accumulation in seeds suggested that the ratio of OBs to cell size was highly positively correlated with oil content [19, 38–40]. Comparing the SOCs of the overexpression and knockdown/knockout lines demonstrated that *BnLPAT* family genes are indispensable for seed oil accumulation in *B. napus*.

RNA-seq results revealed that expression profiles differed among the three seed developmental stages. Herein, we list three major differences between seed oil accumulation in the *BnLPAT2*&*5* overexpression lines and that in the knockdown/knockout lines here: (1) different durations of seed development. The on-going lipid metabolism in the overexpression lines, together with biotic stress in the knockdown and knockout lines at the late seed development stage, indicated that the overexpression of *BnLPAT2* and *BnLPAT5* could prolong seed development, thereby extending the duration of seed oil biosynthesis in *B. napus* [41, 42]. (2) Different expression levels of DEGs. The DEGs involved in lipid metabolism and TAG biosynthesis were significantly up-regulated in the overexpression lines during seed development (Fig. 7a, Additional file 2: Figure S12). Most of the up-regulated DEGs in the TAG assembly pathway in the overexpression lines were down-regulated in the knockdown and knockout lines at the three seed developmental stages. However, the expression profiles of the genes in the TAG-degrading pathway in the overexpression lines were exactly opposite to those in the knockdown and knockout lines. These results might illustrate that TAG biosynthesis was dominant in the *BnLPAT2* and *BnLPAT5* overexpression lines but is recessive in the knockdown and knockout lines [12, 43]. Moreover, the DEGs involved in lipid lipase, such as SDP1, were active in the knockout and knockdown lines (Fig. 7c), showing that lipid catabolism has an indispensable role in the knockout and knockdown lines [44]. (3) Differential regulation related to enriched pathways. GO enrichment analysis showed that in the *Bnlpat2*-RNAi and -Cas9 lines, most DEGs were involved in FA metabolism, which is beneficial to the biosynthesis of long-chain FAs that are further converted into secondary metabolites, and to lipid metabolism, indicating that TAG degradation was promoted [45, 46]. In the *Bnlpat5* knockdown and knockout lines, the significant down-regulation of the DEGs involved in FA/lipid metabolism at 26 DAF was indicative of slow oil biosynthesis [41, 46]. Furthermore, KEGG enrichment analysis showed that the DEGs involved in lipid metabolism might enhance oil biosynthesis in the overexpression lines while promoting oil degradation in the knockdown and knockout lines. In addition, in line with the increase in seed protein and sugar contents, the DEGs of the knockdown and knockout lines were significantly enriched in carbohydrate metabolism, glycan biosynthesis metabolism, and amino acid biosynthesis pathways, thus demonstrating that competitive relationships existed between amino acids/sugar and FA in the use of photosynthesis products [40, 47–49].

### *BnLPAT2* and *BnLPAT5* differentially regulate oil accumulation in* B. napus*

Generally, gene redundancy is considered to be an adaptation of plants, especially polyploid plants, to diverse environments [50]. Thus, not only homologous genes, but also heterologous genes present divergent expression profiles and functions in many plants [19, 51]. In the present study, we found a clear functional difference in oil accumulation between *BnLPAT2* and *BnLPAT5*. The SOCs of the gain-of-function mutants of *BnLPAT2* and *BnLPAT5* showed an average increase of 19.7% and 8.8%, respectively, relative to those of the WT. Furthermore, average decrements of 18.8% and 22.9% were detected in the SOCs of the *Bnlpat2* and *Bnlpat5* knockdown lines, respectively, and of 14.8% and 13.9% in those of the *Bnlpat2*-Cas9 and *Bnlpat5*-Cas9 lines, respectively (Fig. 2d). The differences between the control of SOC by *BnLPAT2* and that by *BnLPAT5* indicated that the diversification of both genes led to the divergence of their expression and function in *B. napus*. Similarly, the overexpression of two *BnLPAT* genes in Arabidopsis, i.e., *BAT1.13* and *BAT1.5*, resulted in an increase of 17% in the seed lipid content of *BAT1.13* transgenic seeds and of 14% in that of *BAT1.5* transgenics [52]. *AtLPAT4* is functionally redundant with *AtLPAT5* in the endoplasmic-reticulum-localized de novo glycerolipid biosynthesis of phospholipids and TAG in Arabidopsis [21]. Furthermore, the SOCs of the *BnLPAT2*&*5* co-overexpression and double mutants did not show additional effects. These results demonstrated that the diverse isoforms of the *LPAT* genes in *B. napus*, which experienced duplication and natural hybridization, were not only functionally redundant but also functionally diverse [17].

Furthermore, the *BnLPAT2* and *BnLPAT5* transgenics showed different expression profiles during seed development. First, the numbers of DEGs and TFs in the *BnLPAT2* transgenic lines were considerably higher than those in the *BnLPAT5* transgenic lines at the three developmental stages (Additional file 2: Figure S9–S10). Second, differential pathways, such as major CHO metabolism and biotic stress, in the *BnLPAT2* transgenic lines were significantly enriched relative to those in the *BnLPAT5* transgenic lines (Fig. 5–6, Additional file 2: Figure S16). These results indicated that overexpression of *BnLPAT2*, but not *BnLPAT5*, favors TAG biosynthesis [46, 53]. Importantly, most DEGs appeared to be involved in the biosynthesis of DAG in LP2 and in PA biosynthesis in LP5 (Fig. 7b), suggesting the different allocation of resources in the two transgenic lines. Consequently, the increase in DAG in LP2 promoted the increased synthesis of storage lipids in mature seeds to maximize the accumulation of seed oils [43, 54]. Collectively, our results illustrated that *BnLPAT2* played a more important role than *BnLPAT5* in regulating oil accumulation. Given that LPAT is the key enzyme in the Kennedy pathway, our future work will emphasize the enzymatic activity of BnLPATs.

## Conclusions

Through overexpression, RNAi, and CRISPR–Cas9 technology, we demonstrated that *BnLPAT2* and *BnLPAT5* are essential for seed oil accumulation. The accumulation of lipid droplets and OBs in the *BnLPAT2* and *BnLPAT5* overexpression lines was accompanied by decrements in sugar and protein contents. Moreover, in *B. napus*, *BnLPAT2* appeared to contribute more to SOC than *BnLPAT5*. RNA-seq analysis also revealed that *BnLPAT2* preferentially promoted diacylglycerol synthesis for the accumulation of SOC, whereas *BnLPAT5* tended to boost PA synthesis for membrane lipid generation. Therefore, *BnLPAT2* and *BnLPAT5* could jointly but differently promote seed oil accumulation in *B. napus*. This study provides valuable insights into the promotion of the SOC of the seeds of *B. napus* and may thus facilitate the high production of vegetable oil in the future.

## Methods

### Plant materials and sampling

In this study, the semi-winter rapeseed pure variety ‘J2016’ was taken as the transformation receptor to obtain transgenic lines as described in previous studies [32, 35]. The transgenic T1 plants were identified and planted in Wuhan for the collection of T2 seeds in May 2018. The T3 seeds were harvested from successive summer generations in Linxia (Gansu) in September 2018. Then, the T2 and T3 seeds were planted in Wuhan to obtain the corresponding T2 and T3 transgenic plants for further experiments.

The flowering times of the main and secondary inflorescences of each line were marked, and developing seeds were collected from T3 progenies at 14 DAF (the early stage for oil accumulation), 20 DAF, 26 DAF (the rapid stage for oil accumulation), 32 DAF, and 38 DAF (the slow stage of oil accumulation) for further experiments. The sampled seeds were quickly frozen in liquid nitrogen and then stored at −80 ℃. Mature seeds from all the T2 and T3 progenies were also collected for further experiments.

### Molecular identification of transgenic-positive and mutant plants

The CDS fragments of *BnaA07g17400D* and *BnaC05g34170D* were cloned into pCMABIA-1303 for *BnLPAT2* and *BnLPAT5* overexpression. Two conserved regions (i.e., 364–839 and 575–986 nt) were designed in *BnaA07g17400D* for *BnLPAT2* knockdown (namely, LP2-R1 and LP2-R2), and two conserved regions (i.e., 220–691 and 579–951 nt) were designed in *BnaC05g34170D* for *BnLPAT5* knockdown (namely, LP5-R3 and LP5-R4) as previously described [35]. For OE and RNAi lines, the transgenic-positive plants of T2 and T3 progenies were identified through PCR amplification with the primers listed in Additional file 1: Table S8. For knockout lines, mutants were identified through PCR amplification and Sanger sequencing. For the co-overexpression lines of *BnLPAT2* and *BnLPAT5*, transgenic-positive LP2&LP5-OE plants were identified as LP2-OE and LP5-OE through further transcript level identification. BnaA07g17400D-RT-F/R and BnaC05g34170D-RT-F/R primers were used for the determination of the relative expression levels of *BnLPAT2* and *BnLPAT5*. RT-qPCR was carried out by using SYBR Green Realtime PCR Master Mix (YEASEN, China) and the Stepone Plus platform (ABI, America) in accordance with the provided protocol with three technical replicates for each line. Relative gene expression level was calculated through the ^ΔΔ^Ct method, and the designed primers are listed in Additional file 1: Table S9. The *BnTIF4* gene was used as the reference gene (ACTIN).

### Seed oil, sugar, and protein extraction, examination, and analysis

Mature seeds were dried through passerillage, and developing seeds were dried by using a freeze dryer (SCIENTZ-10ND, China). Afterward, the total oil content was extracted in accordance with a previous study [32]. SOC was analyzed through gas chromatography with Agilent 7890A (Agilent, USA) as previously described [32].

The tissues of dry mature seeds were ground into powder in liquid nitrogen, and approximately 100 mg of tissue powder was used for sugar and protein detection with three biological replicates for each sample. The total sugar was extracted in accordance with the protocol of a total sugar content detection kit (Solarbio, China), then subjected to absorbance examination at A540nm with three technical replicates for each sample. The total protein was extracted by using a plant protein extraction kit (Solarbio, China) then detected by using a Bradford protein assay kit (Solarbio, China). Afterward, the absorbance of the total protein solution was examined at A595nm with three technical replicates for each sample.

### Lipid droplet inspection under laser confocal fluorescence microscopy

Approximately three blooming rapeseed flowers were collected from each plant at dusk and placed on ice, and the pollens were absolutely matured at sunset. Next, EP tubes containing pollens and deionized water were centrifuged at 3000 rpm for 2 min. Then, the supernatant was pipetted off to collect the pollens at the bottom of the tubes. After Nile Red staining in the dark for approximately 10 min, the lipid droplets in the mature pollens were inspected at the excitation wavelength of 633 nm by using the laser confocal fluorescence microscope FV1000 (Olympus, Japan). Images were captured from several visual fields of each sample.

### TEM examination and analysis

For TEM examination and analysis, dicotyledons were isolated from dried mature seeds in deionized water and fixed in 2.5% glutaraldehyde then vacuumed by using a vacuum drying oven (JINGHONG, China). The dicotyledons were then fixed again in 1% gallic acid and 0.1 mol/L phosphate buffer solution for 2 h and rinsed with phosphate buffer solution three times for 15 min each time. Next, the dicotyledons were dehydrated in a gradient series of alcohol (i.e., 50%–70%–80%–90%–95%–100%–100%) for 15 min each time, then infiltrated with a mixed infiltration solution (i.e., acetone:812 embedding agents = 1:1) overnight and polymerized for 48 h at 60 ℃ for embedding. Subsequently, 60–80 nm ultrathin slices were obtained by using Leica UC7 (Leica, Germany) then stained with lead citrate and uranyl acetate for 15 min. Images were captured by using TEM TECNAI G2 20 TWIN (FEI, America) under different magnifications. Cell and protein sizes were evaluated by using Image J. All the PBs in one cell were counted, and at least three cells were counted for each sample.

### Seed morphology scanning and TSW analysis

For seed morphology scanning, approximately 500 mg of dry mature seeds with three biological replicates for each line were weighed and scanned by using a SC-G automatic seed tester and TSW analyzer (Wseen, China) as previously described [32]. The seeds being scanned were arranged in accordance with the area (i.e., from large to small) of each sample. TSW was calculated as total weight/total number of seeds being scanned ×1000 for each experiment.

### Library construction, Illumina sequencing, read mapping, and DEG analysis

Each sample was subjected to total RNA extraction, library construction, Illumina sequencing, read mapping, and DEG analysis as described in a previous study with minor modifications [55]. Briefly, total RNA was extracted from approximately 100 mg of seeds with three biological replicates for each sample by using TRIzol^®^ Reagent in accordance with the manufacturer’s instructions (Invitrogen, USA). Next, the RNA-seq library was prepared by following the instructions of the TruSeq™ RNA sample preparation Kit (Illumina, USA) then sequenced on an Illumina HiSeq X Ten platform (2 × 150 bp read length). The generated raw reads were trimmed and subjected to quality control by using SeqPrep (https://github.com/jstjohn/SeqPrep) and Sickle (https://github.com/najoshi/sickle) with default parameters. The clean reads were then separately mapped to the *B. napus* reference genome in orientation mode by using TopHat software (http://tophat.cbcb.umd.edu/, version2.0.0) [56]. For interlibrary comparisons, the expression level of each transcript was calculated in accordance with the FRKM method by utilizing the RSEM package [57], and the genes with FPKM ≥ 1 were identified as expressed. DEG analysis was conducted on all samples with the R package DEseq [58], and genes exhibiting a difference of at least a twofold change with corrected P-value ≤ 0.05 in transgenic lines relative to those in the WT were regarded as significantly differentially expressed.

### Functional categorization and analysis of DEGs

Functional categorization, including GO and KEGG terms, was conducted with Goatools (https://github.com/tanghaibao/Goatools) and KOBAS (http://kobas.cbi.pku.edu.cn/home.do). In addition, GO enrichment analysis was conducted by applying Cytoscape in accordance with a previous study with minor modifications [41]. Briefly, the DEGs were loaded into Cytoscape by BiNGO [59]. The P-value was calculated and corrected via the Benjamini–Hochberg error correction method, and GO terms with corrected P-value ≤ 0.05 were considered to be significantly enriched. Subsequently, the DEGs were loaded into Cytoscape again with EnrichmentMap to unravel the detailed GO enrichment pathways. The pathway enrichment of the DEGs was conducted by using MapMan (v3.5.1R2) in reference to the mapping of *Brassica*_*napus*.annotation_v5.cds.fa.gz.

## Supplementary Information

**Additional file 1: Table S1. **Transformation efficiency of different lines. **Table S2.** Detailed information of RNA-seq samples. **Table S3.** Summary of read data generated, quality control and mapping on the B. napus genome for different samples. **Table S4. **Numbers of genes expressed at different expression levels at the different seed development stages. **Table S5. **Numbers of commonly and specifically expressed DEGs at each stage of seed development in *BnLPAT2* and *BnLPAT5* transgenic lines. **Table S6. **Detailed GO enrichments of all DEGs at different transgenic lines during seed development. **Table S7. **Enriched KEGG pathways involved in metabolism for ten subclusters of all DEGs. **Table S8. **Enriched KEGG pathways involved in basic metabolism at late seed development of different transgenic lines. **Table S9. **Primers used in present study.

**Additional file 2: Figure S1.** Sanger sequencing for Cas9 cleavage activity in other rapeseed varieties. **Figure S2.** TSW of the mature seeds in all *BnLPAT2* and *BnLPAT5* transgenic lines of T2 generation. **Figure S3.** Microstructure of the mature cotyledons in all *BnLPAT2* and *BnLPAT5* transgenic lines of T2 generation. **Figure S4. **The histogram of the cell size and protein body in *BnLPAT2* and *BnLPAT5* transgenic lines. **Figure S5. **SOC during the seed development in *BnLPAT2* and *BnLPAT5* transgenic lines. **Figure S6.** PCC analysis between the *BnLPAT2* and *BnLPAT5* transgenic lines at three seed development stages. **Figure S7. **PCC analysis among the three biological replicates of each tissue sample in *BnLPAT2* lines. **Figure S8. **PCC analysis among the three biological replicates of each tissue sample in *BnLPAT5* lines. **Figure S9. **Correlation between expression profiles of selected genes from RNA-seq and RT-qPCR analysis. **Figure S10. **Numbers of up-regulated and down-regulated genes at each stage of seed development in all *BnLPAT2* and *BnLPAT5* transgenic lines. **Figure S11. **Venn diagrams summarizing the DEG numbers detected in *BnLPAT2* and *BnLPAT5* lines at different seed development stages. **Figure S12. **Numbers of TF families during seed development in *BnLPAT2* and *BnLPAT5* lines. **Figure S13. **Heatmap of DEGs in photosynthesis-antenna proteins and cutin, suberin and wax biosynthesis pathways. **Figure S14. **GO enrichment analysis of DEGs at all the stages of seed developments in *BnLPAT2* and/or *BnLPAT5* lines. **Figure S15. **Enriched GO terms at different stages of seed development in *BnLPAT2* and *BnLPAT5 *transgenic lines. **Figure S16. **Differential expression of the representative genes involved in lipid metabolism during seed development. **Figure S17. **Metabolic pathways and biotic stress pathways showing differential expression in *BnLPAT2* and *BnLPAT5* overexpression and knockout lines at 26 DAF and 38DAF.

## Data Availability

Additional files 1–2: additional material of “Lysophosphatidic acid acyltransferase 2 and 5 commonly, but differently, promote seed oil accumulation in Brassica napus.” The raw reads of sequencing data used by this study were deposited in the SRA database of NCBI (SRR14562210-SRR14562272).

## Abbreviations

LPAT2: Lysophosphatidic acid acyltransferase 2
LPAT5: Lysophosphatidic acid acyltransferase 5
SOC: Seed oil content
TAG: Triacylglycerol
DAG: Diacylglycerol
PA: Phosphatidic acid
DEG: Differentially expressed gene
TSW: Thousand seed weight
TEM: Transmission electron microscope
DAF: Day after flowering
OB: Oil body
PB: Protein body
EC: Early cluster
LC: Late cluster
MC: Middle cluster
LP2-G1: GRNA1-mediated *Bnlpat2* knockout line
LP2-G2: GRNA2-mediated *Bnlpat2* knockout line
LP2-G3: GRNA3-mediated *Bnlpat2* knockout line
LP5-G4: GRNA4-mediated *Bnlpat5* knockout line
LP2-OE: *BnLPAT2* Overexpression line
LP5-OE: *BnLPAT5* Overexpression line
LP2-R1: RNAi1-mediated *Bnlpat2* knockdown line
LP2-R2: RNAi2-mediated *Bnlpat2* knockdown line
LP5-R3: RNAi3-mediated *Bnlpat5* knockdown line
LP5-R4: RNAi4-mediated *Bnlpat5* knockdown line
G3P: Glycerol 3-phosphate
GPAT: Glycerol-3-phosphate acyltransferase
LPAT: 2-Lysophosphatidic acid acyltransferase
DGAT: Diacylglycerol acyltransferase
PC: Phosphatidylcholine
SDP1: Sugar-dependent1
WT: Wild type
LTPs: Lipid transfer proteins
