# Supplementary material for: Lysophosphatidic acid acyltransferase 2 and 5 commonly, but differently, promote seed oil accumulation in Brassica napus
Source: Biotechnol Biofuels Bioprod. 2022 Aug 12;15:83. doi: 10.1186/s13068-022-02182-2 (PMC9375321; doi:10.1186/s13068-022-02182-2)
Supplement: Supplementary file 2 — Additional file 2: Figure S1. Sanger sequencing for Cas9 cleavage activity in other rapeseed varieties. Figure S2. TSW of the mature seeds in all BnLPAT2 and BnLPAT5 transgenic lines of T2 generation. Figure S3. Microstructure of the mature cotyledons in all BnLPAT2 and BnLPAT5 transgenic lines of T2 generation. Figure S4. The histogram of the cell size and protein body in BnLPAT2 and BnLPAT5 transgenic lines. Figure S5. SOC during the seed development in BnLPAT2 and BnLPAT5 transgenic lines. Figure S6. PCC analysis between the BnLPAT2 and BnLPAT5 transgenic lines at three seed development stages. Figure S7. PCC analysis among the three biological replicates of each tissue sample in BnLPAT2 lines. Figure S8. PCC analysis among the three biological replicates of each tissue sample in BnLPAT5 lines. Figure S9. Correlation between expression profiles of selected genes from RNA-seq and RT-qPCR analysis. Figure S10. Numbers of up-regulated and down-regulated genes at each stage of seed development in all BnLPAT2 and BnLPAT5 transgenic lines. Figure S11. Venn diagrams summarizing the DEG numbers detected in BnLPAT2 and BnLPAT5 lines at different seed development stages. Figure S12. Numbers of TF families during seed development in BnLPAT2 and BnLPAT5 lines. Figure S13. Heatmap of DEGs in photosynthesis-antenna proteins and cutin, suberin and wax biosynthesis pathways. Figure S14. GO enrichment analysis of DEGs at all the stages of seed developments in BnLPAT2 and/or BnLPAT5 lines. Figure S15. Enriched GO terms at different stages of seed development in BnLPAT2 and BnLPAT5 transgenic lines. Figure S16. Differential expression of the representative genes involved in lipid metabolism during seed development. Figure S17. Metabolic pathways and biotic stress pathways showing differential expression in BnLPAT2 and BnLPAT5 overexpression and knockout lines at 26 DAF and 38DAF. [file 13068_2022_2182_MOESM2_ESM.doc]

**Additional file 2**

**Figure S1.** Sanger sequencing for Cas9 cleavage activity in other rapeseed varieties.

**Figure S2.** TSW of the mature seeds in all *BnLPAT2* and *BnLPAT5* transgenic lines of T2 generation.

**Figure S3.** Microstructure of the mature cotyledons in all *BnLPAT2* and *BnLPAT5* transgenic lines of T2 generation.

**Figure S4.** The histogram of the cell size and protein body in *BnLPAT2* and *BnLPAT5* transgenic lines.

**Figure S5.** SOC during the seed development in *BnLPAT2* and *BnLPAT5* transgenic lines.

**Figure S6.** PCC analysis between the *BnLPAT2* and *BnLPAT5* transgenic lines at three seed development stages.

**Figure S7.** PCC analysis among the three biological replicates of each tissue sample in *BnLPAT2* lines.

**Figure S8.** PCC analysis among the three biological replicates of each tissue sample in *BnLPAT5* lines.

**Figure S9.** Correlation between expression profiles of selected genes from RNA-seq and RT-qPCR analysis.

**Figure S10.** Numbers of up-regulated and down-regulated genes at each stage of seed development in all *BnLPAT2* and *BnLPAT5* transgenic lines.

**Figure S11.** Venn diagrams summarizing the DEG numbers detected in *BnLPAT2* and *BnLPAT5* lines at different seed development stages.

**Figure S12.** Numbers of TF families during seed development in *BnLPAT2* and *BnLPAT5* lines.

**Figure S13.** Heatmap of DEGs in photosynthesis-antenna proteins and cutin, suberine and wax biosynthesis pathways.

**Figure S14.** GO enrichment analysis of DEGs at all the stages of seed developments in *BnLPAT2* and/or *BnLPAT5* lines.

**Figure S15.** Enriched GO terms at different stages of seed development in *BnLPAT2* and *BnLPAT5* transgenic lines.

**Figure S16.** Differential expression of the representative genes involved in lipid metabolism during seed development.

**Figure S17.** Metabolic pathways and biotic stress pathways showing differential expression in *BnLPAT2* and *BnLPAT5* overexpression and knockout lines at 26 DAF and 38DAF.


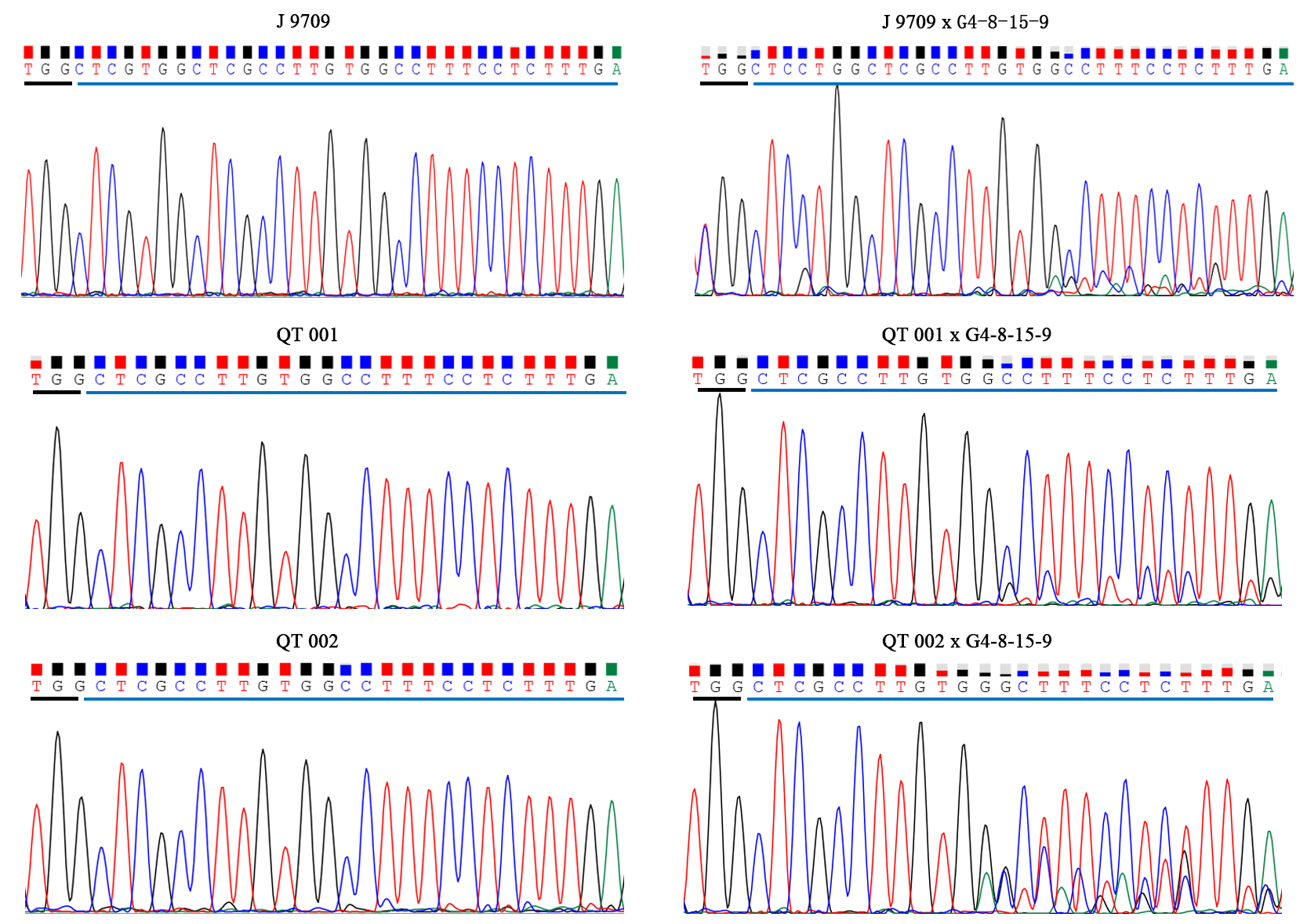


**Figure S1.** Sanger sequencing for Cas9 cleavage activity in other rapeseed varieties. The gene sequences under black line represent PAM region while under blue line represent on-target region.


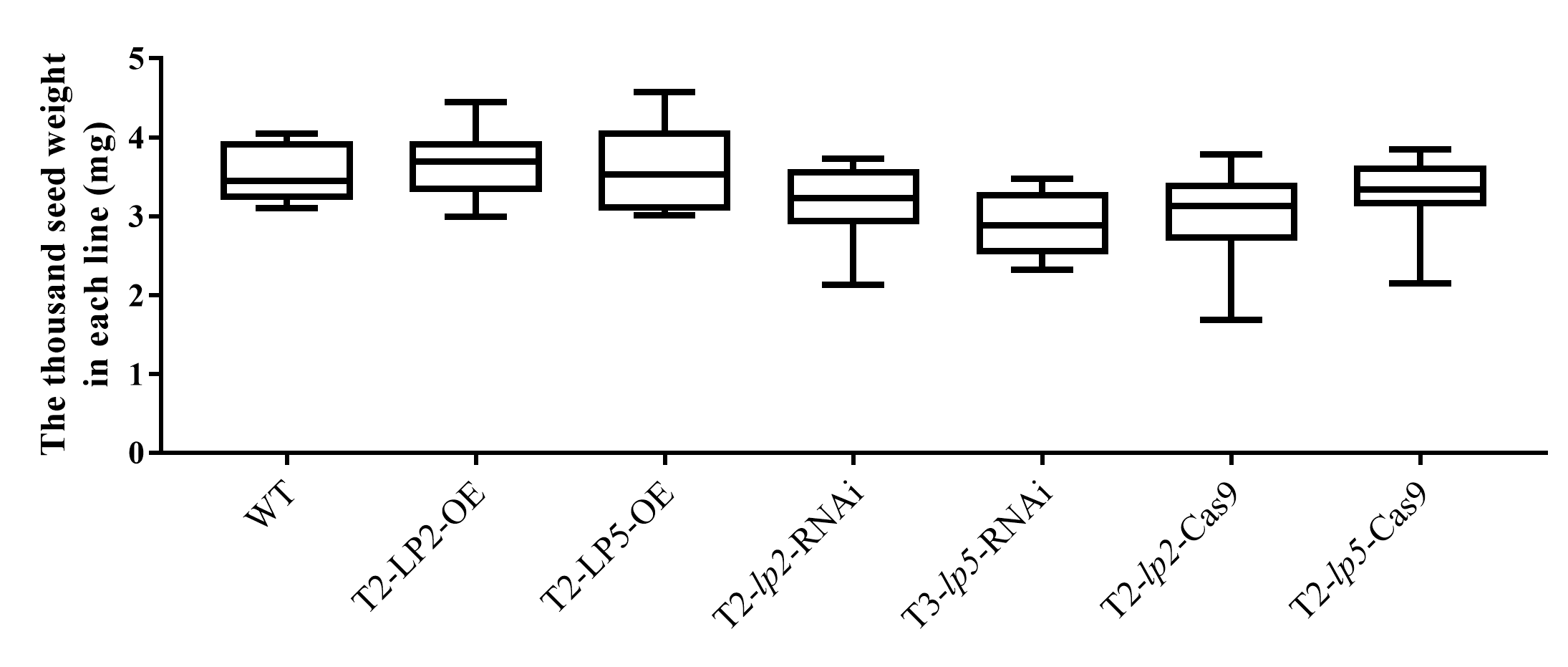


**Figure S2.** TSW of the mature seeds in all *BnLPAT2* and *BnLPAT5* transgenic lines of T2 generation.


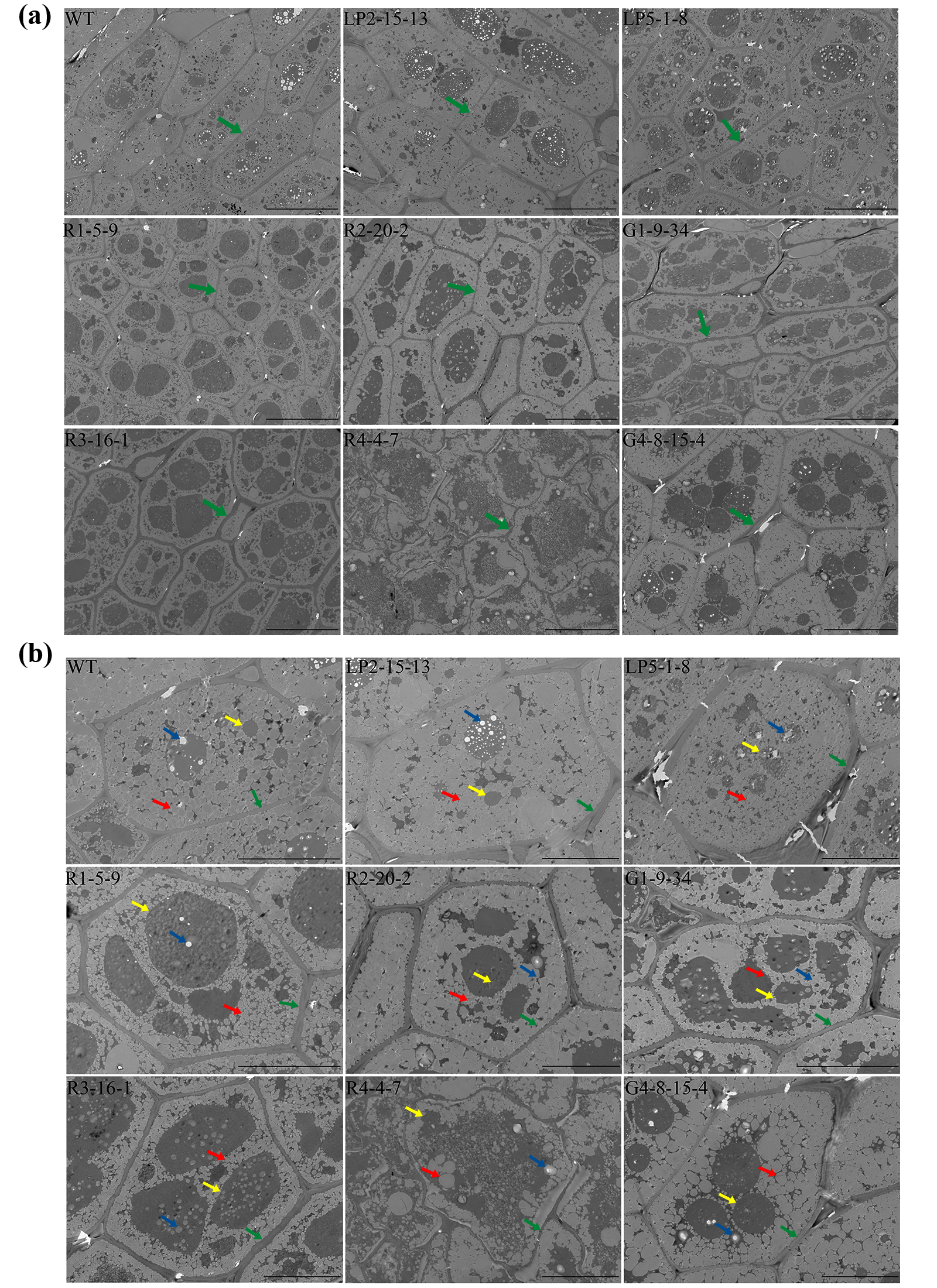


**Figure S3.** Microstructure of the mature cotyledons in all *BnLPAT2* and *BnLPAT5* transgenic lines of T2 progeny. (a) The microstructure of cotyledon in mature seeds of different *BnLPAT2* and *BnLPAT5* transgenic lines in T2 progeny. Bar=20 μm. (b) The microstructure of cotyledon in mature seeds of different *BnLPAT2* and *BnLPAT5* transgenic lines in T2 progeny. Bar=10 μm. The green arrows indicate the cell wall. The red arrows indicate OB. The yellow arrows indicate protein body. The blue arrows indicate starch.


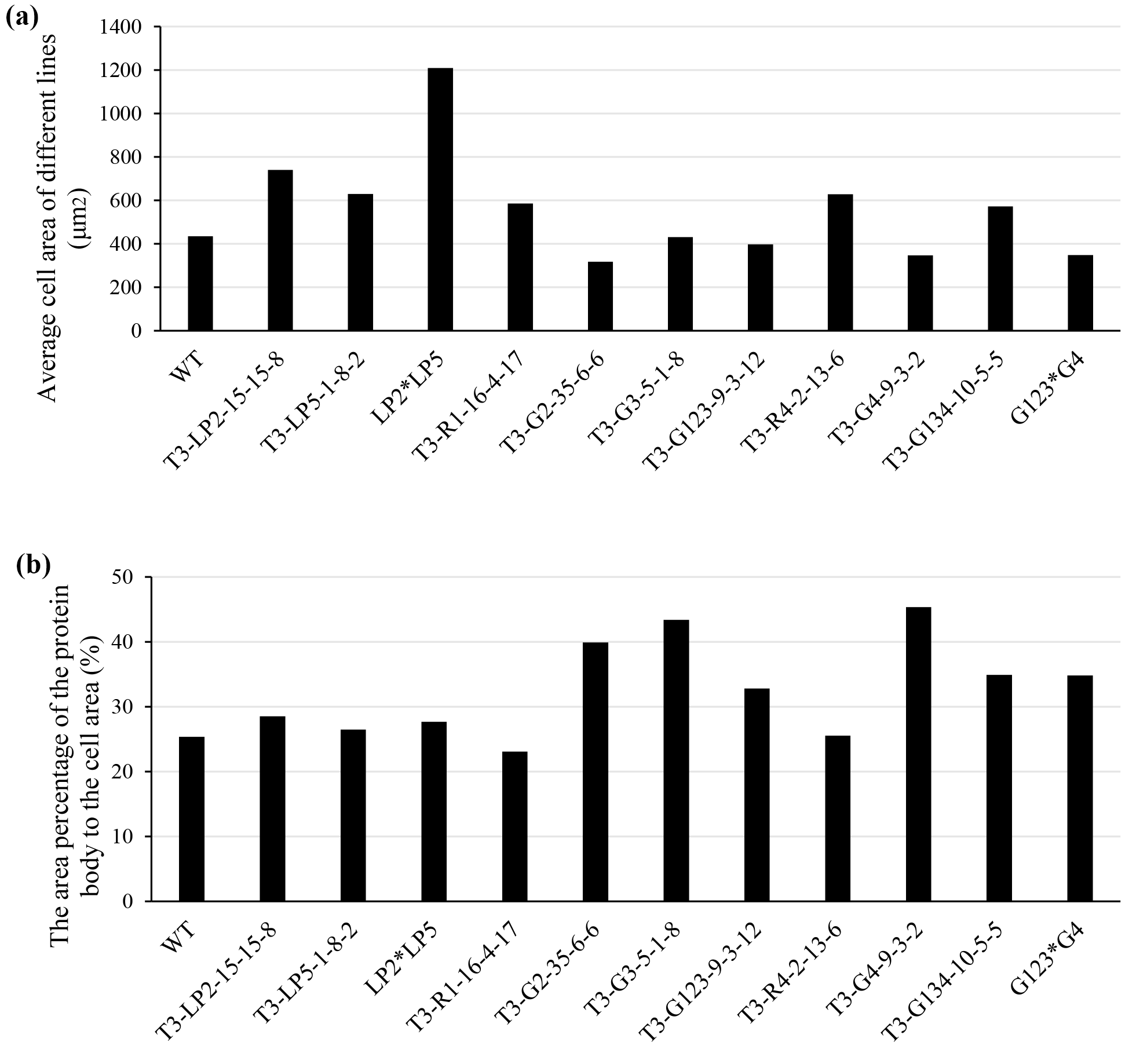


**Figure S4.** The histogram of the cell size and protein body in *BnLPAT2* and *BnLPAT5* transgenic lines. (a) The area ratio of total oil bodies and the average size of each oil body in all *BnLPAT2* and *BnLPAT5* transgenic lines. (b) The area percentage of the protein bodies to the cell area in each *BnLPAT2* and *BnLPAT5* transgenic lines.


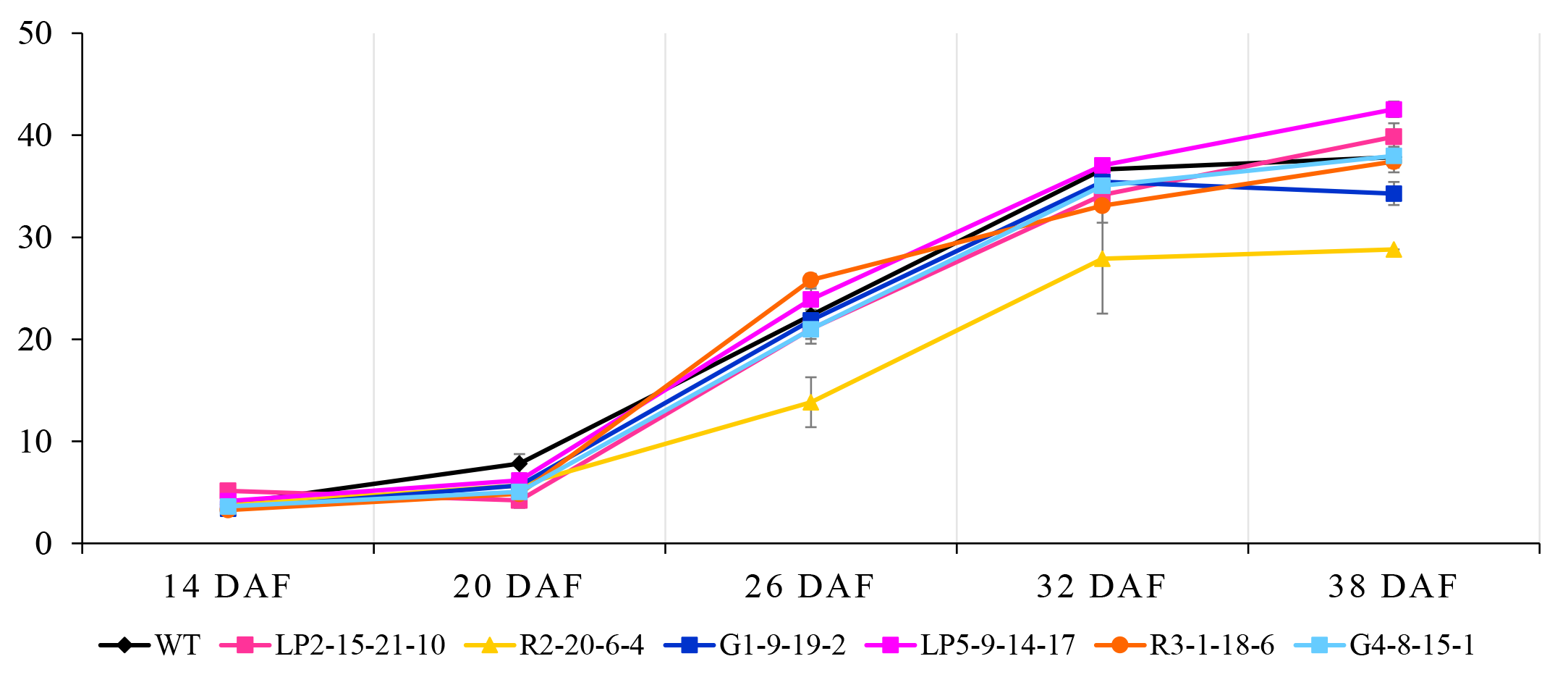


**Figure S5.** SOC during the seed development in *BnLPAT2* and *BnLPAT5* transgenic lines.


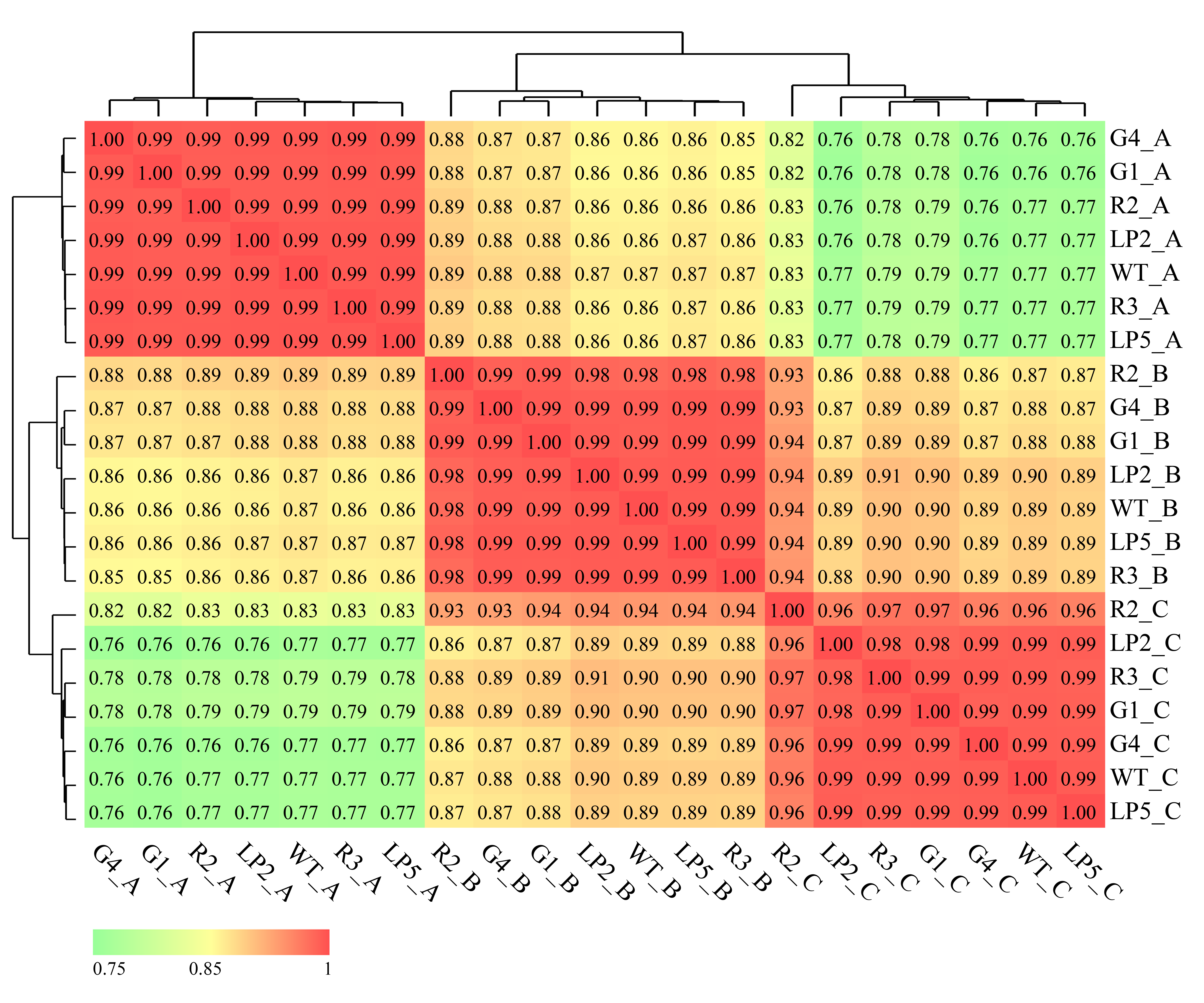


**Figure S6.** PCC analysis between transcriptomes of *BnLPAT2* and *BnLPAT5* transgenic lines at three seed development stages. SCC among *BnLPAT2* lines and *BnLPAT5* lines is shown. A, B and C represent 14DAF, 26DAF and 38DAF.


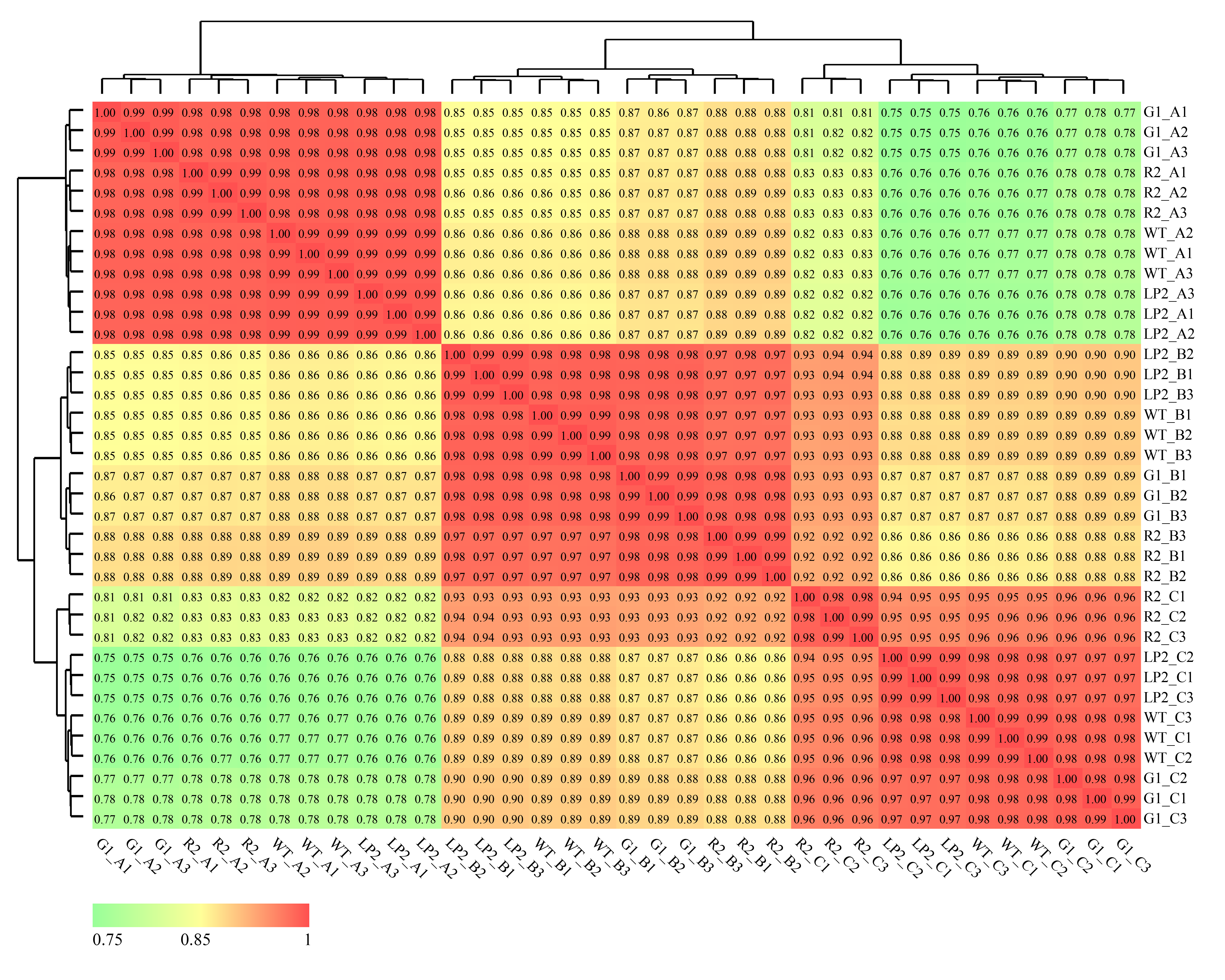


**Figure S7.** PCC analysis among the three biological replicates of each tissue sample in *BnLPAT2* lines. SCC among the replicates of *BnLPAT2* lines is shown. A represents 14DAF; B represents 26DAF; C represents 38DAF. 1-3 represent three biological replicates of each sample.


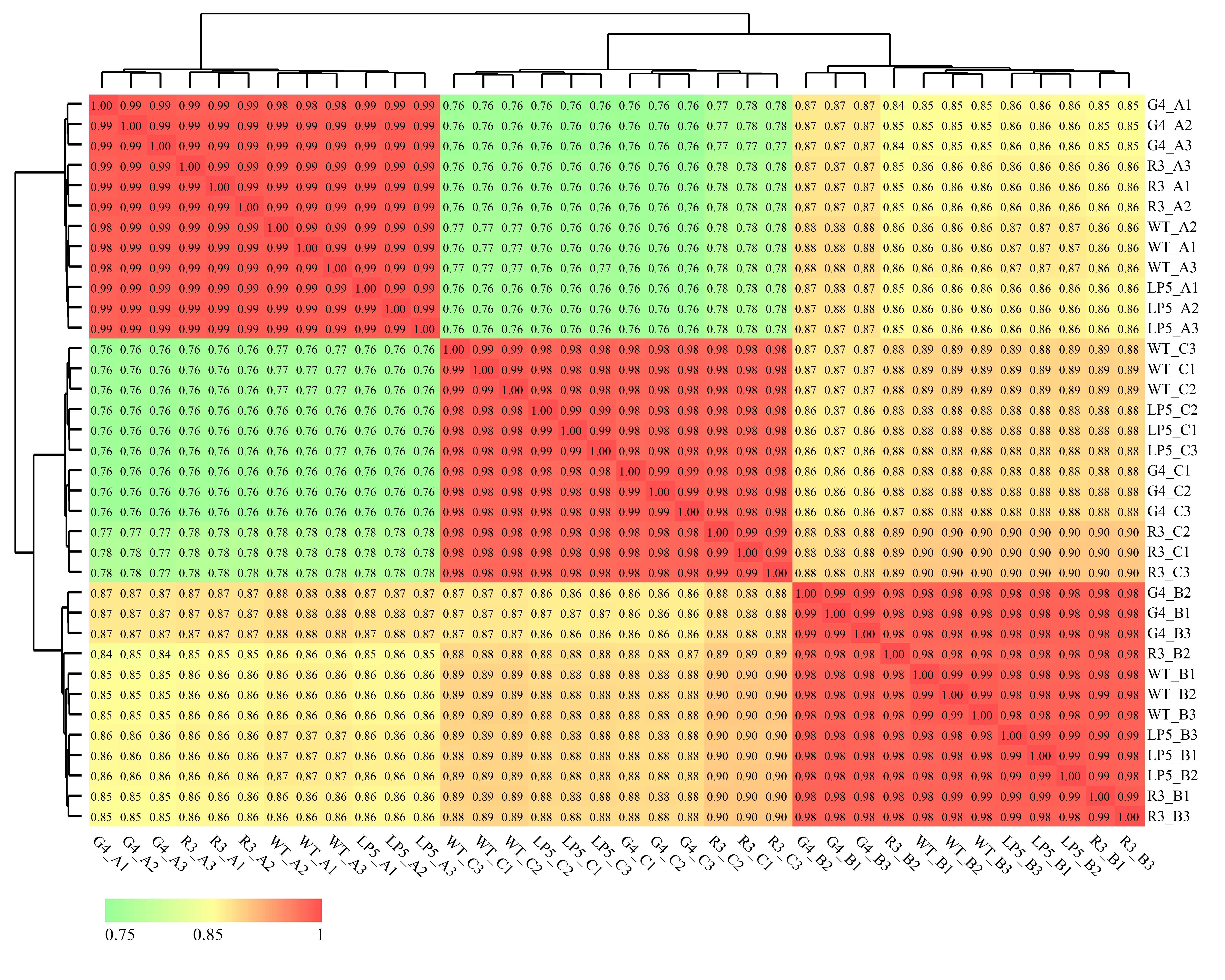


**Figure S8.** PCC analysis among the three biological replicates of each tissue sample in *BnLPAT5* lines. SCC among the replicates of *BnLPAT5* lines is shown. A represents 14DAF; B represents 26DAF; C represents 38DAF. 1-3 represent three biological replicates of each sample.


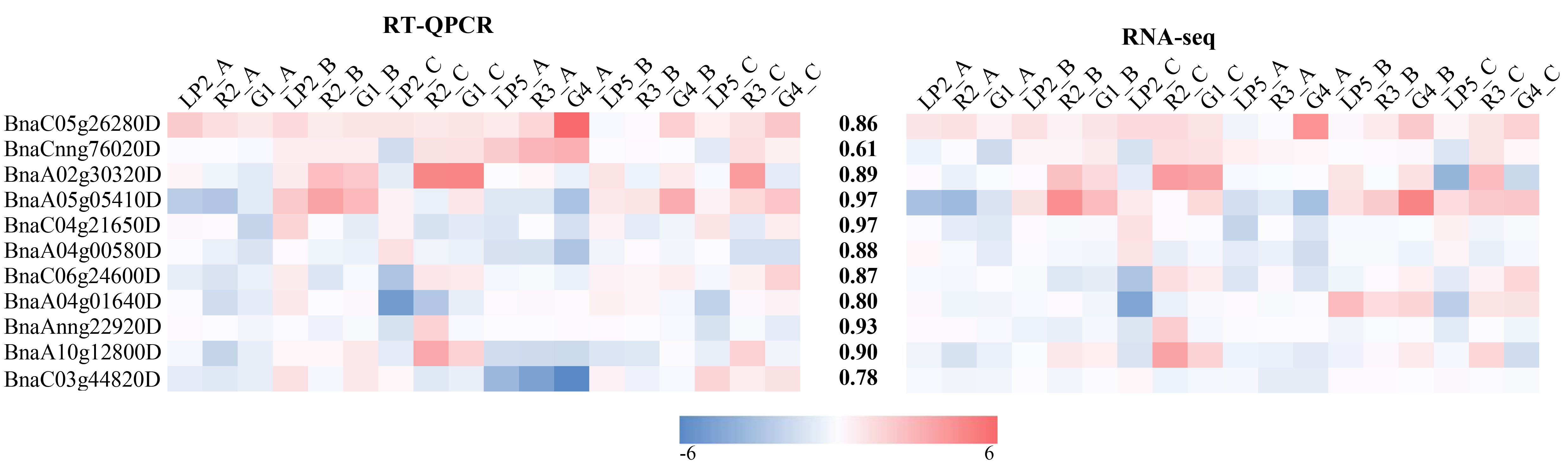


**Figure S9.** Correlation between expression profiles of selected genes obtained from RNA-seq and RT-PCR analysis. Heatmaps represent expression profiles of selected genes (labelled on right) obtained from RT-qPCR (right) and RNA-seq (left) analysis. The color scale at the bottom represents Z-score. The values between the two heatmaps represent correlation value between the expression profiles obtained from RNA-seq and RT-qPCR analysis for each gene.


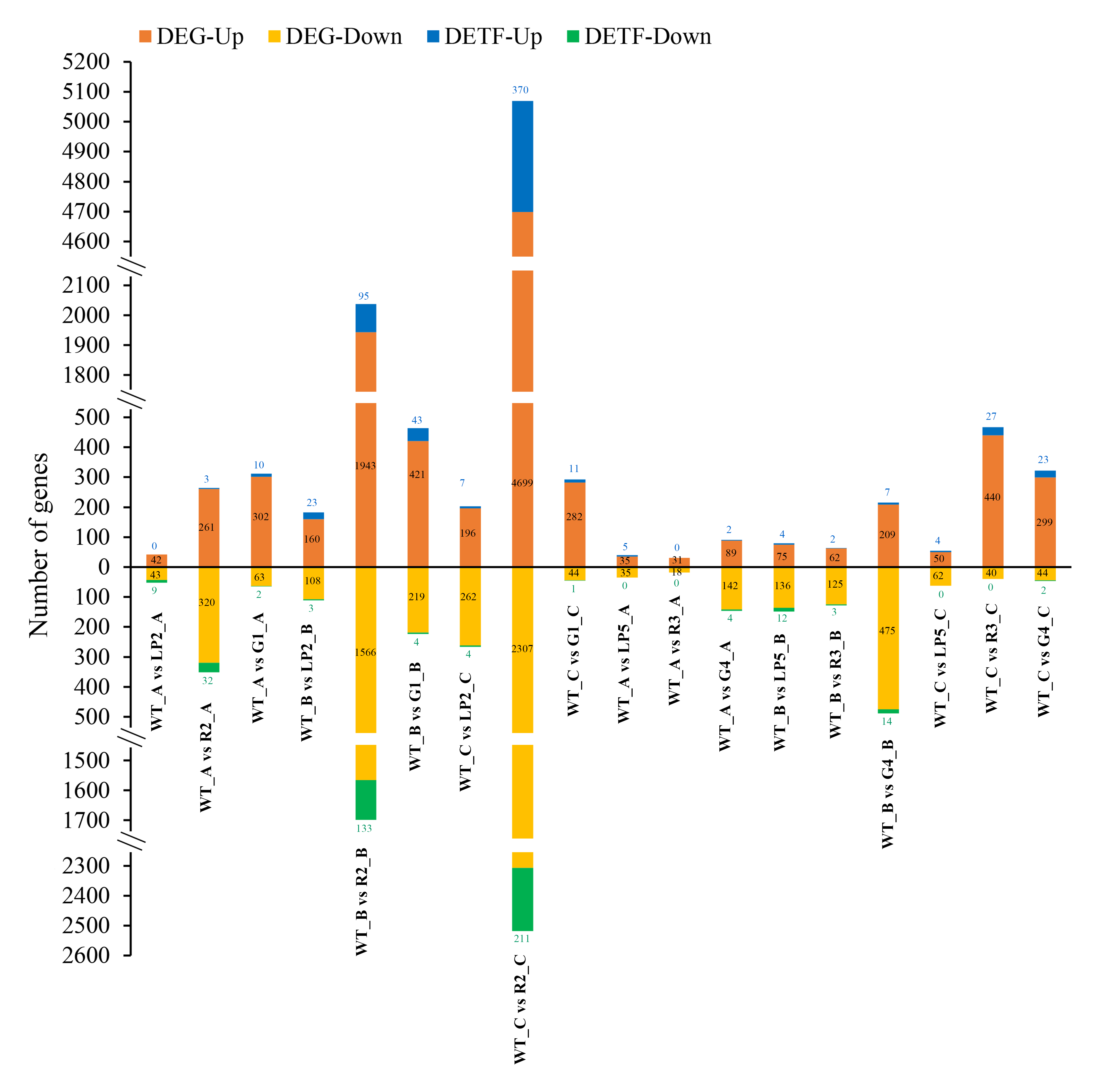


**Figure S10.** Number of up-regulated and down-regulated genes at each stage of seed development in all *BnLPAT2* and *BnLPAT5* transgenic lines. The number of TFs up- or down-regulated at each stage of seed development is given.


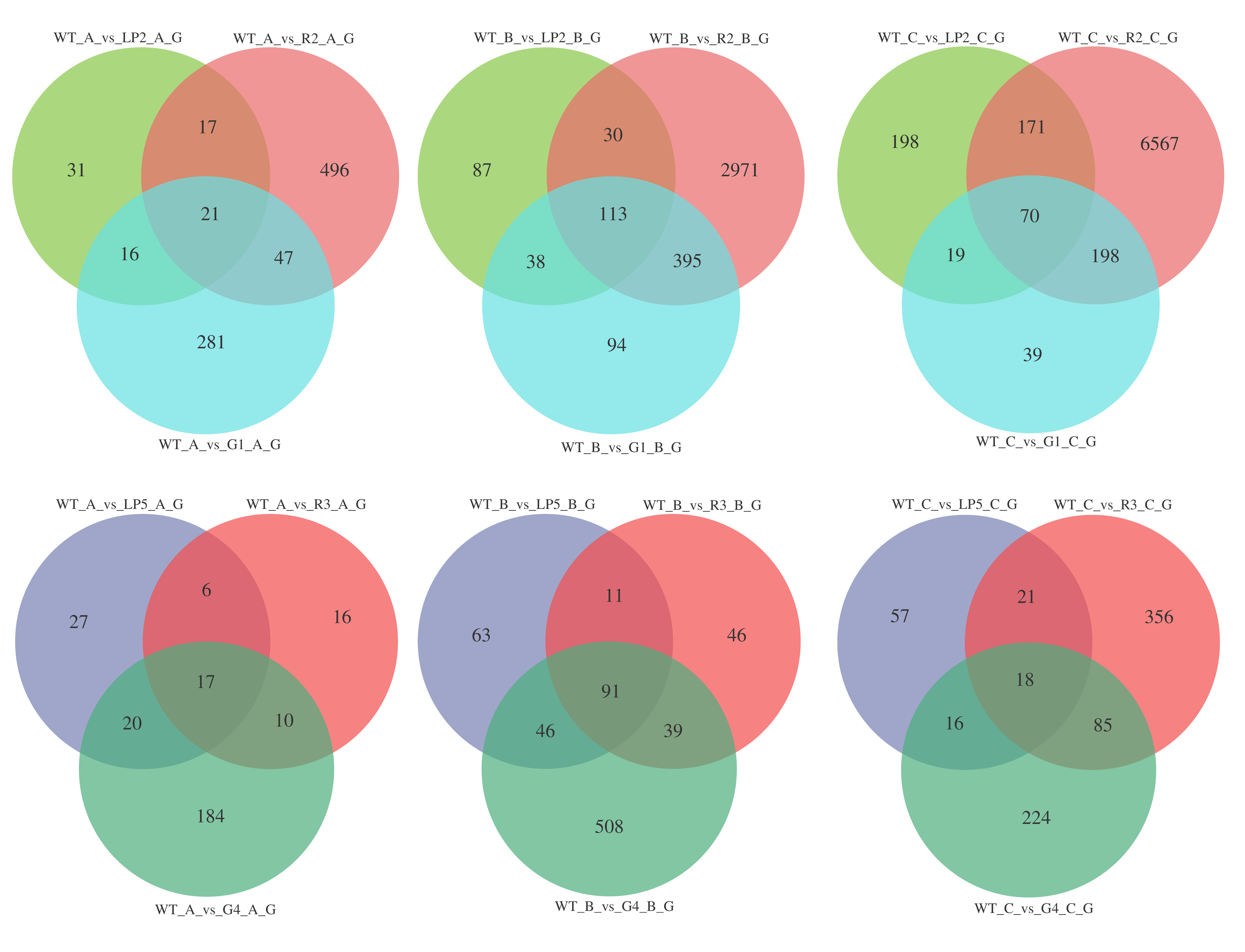


**Figure S11.** Venn diagrams summarizing the DEG number detected in *BnLPAT2* and *BnLPAT5* lines at different seed development stages.


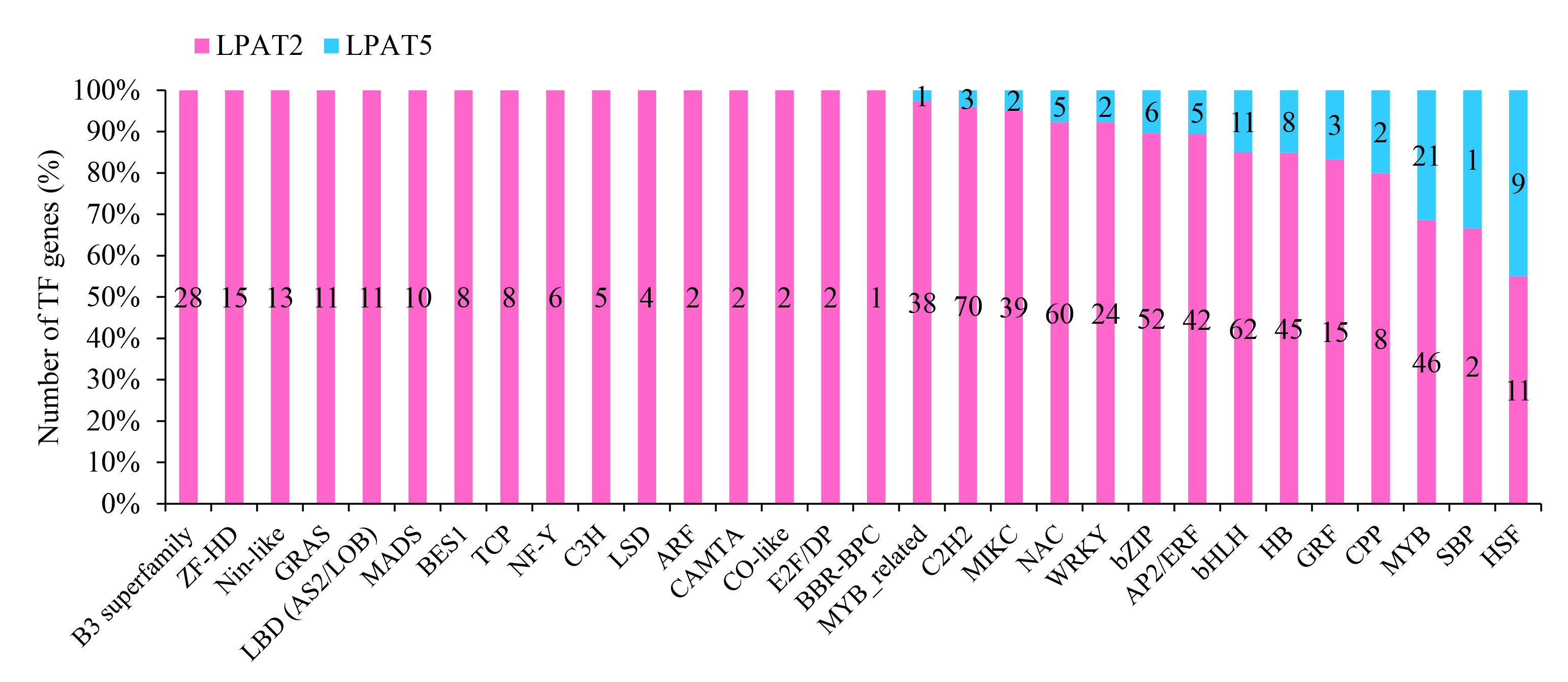


**Figure S12.** Number of TF families during seed development in *BnLPAT2* and *BnLPAT5* lines. Values labeled in column diagram represent the gene number of the corresponded TF family. Red diagram indicates the TF family in *BnLPAT2* lines, and the blue diagram indicates the TF family in *BnLPAT5* lines.


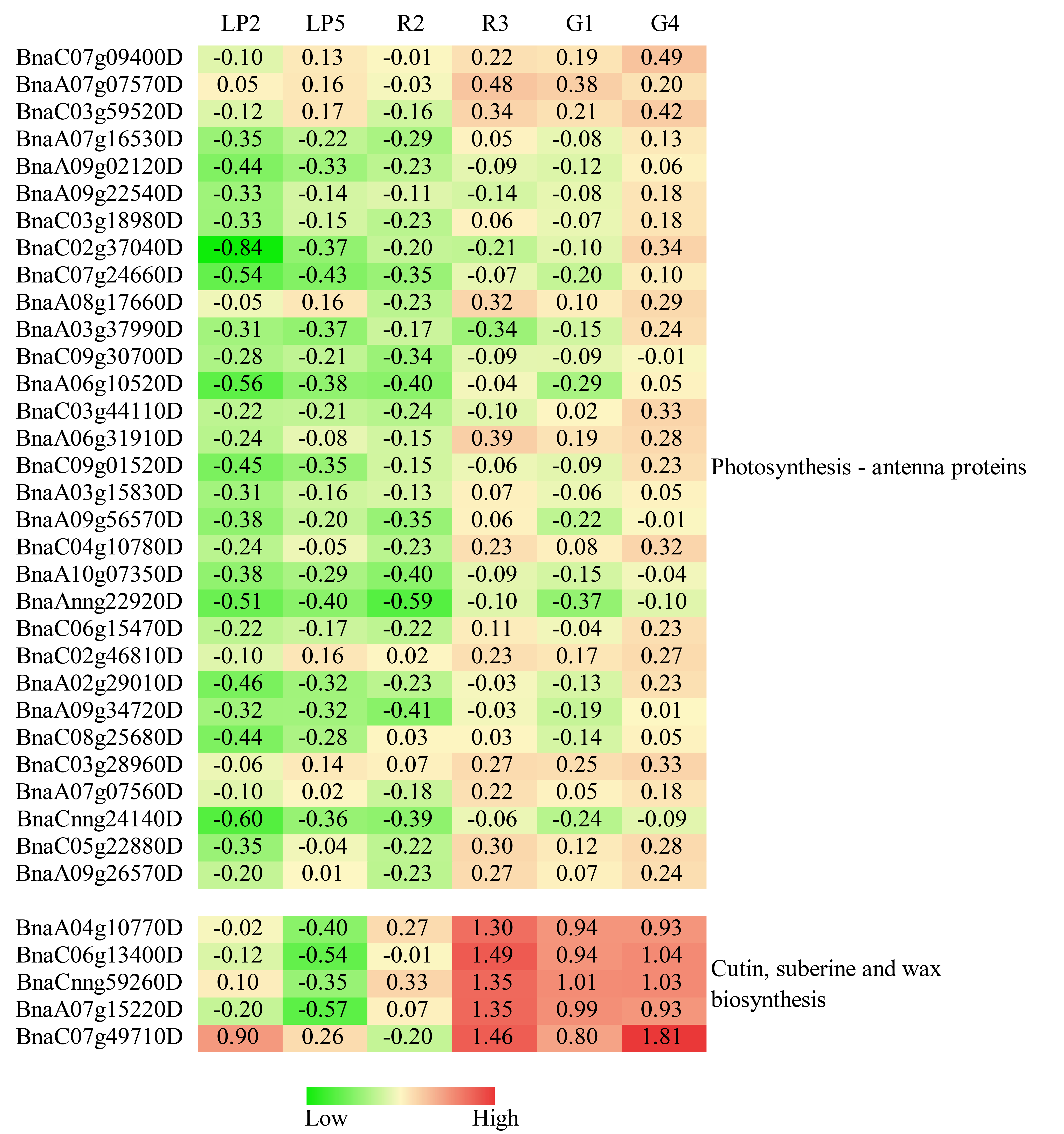
**Figure S13.** Heatmap of DEGs in photosynthesis - antenna proteins and cutin, suberine and wax biosynthesis pathways.


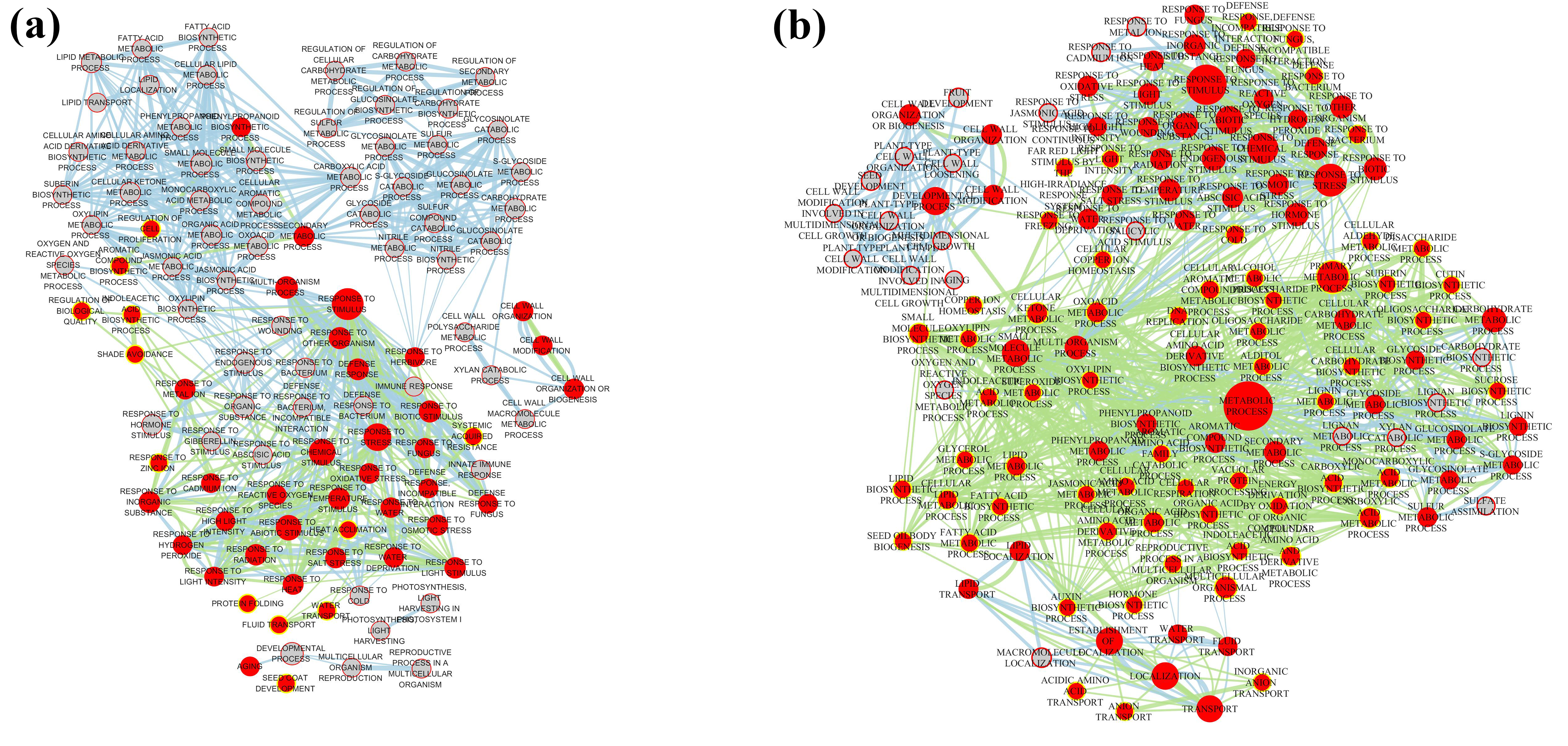


**Figure S14.** GO enrichment map of DEGs at all the stages of seed developments in *BnLPAT2* and/or *BnLPAT5* lines. Significantly enriched GO terms (biological process) were overlapped to highlight the terms specifically or commonly in *BnLPAT2* and *BnLPAT5* lines.

**
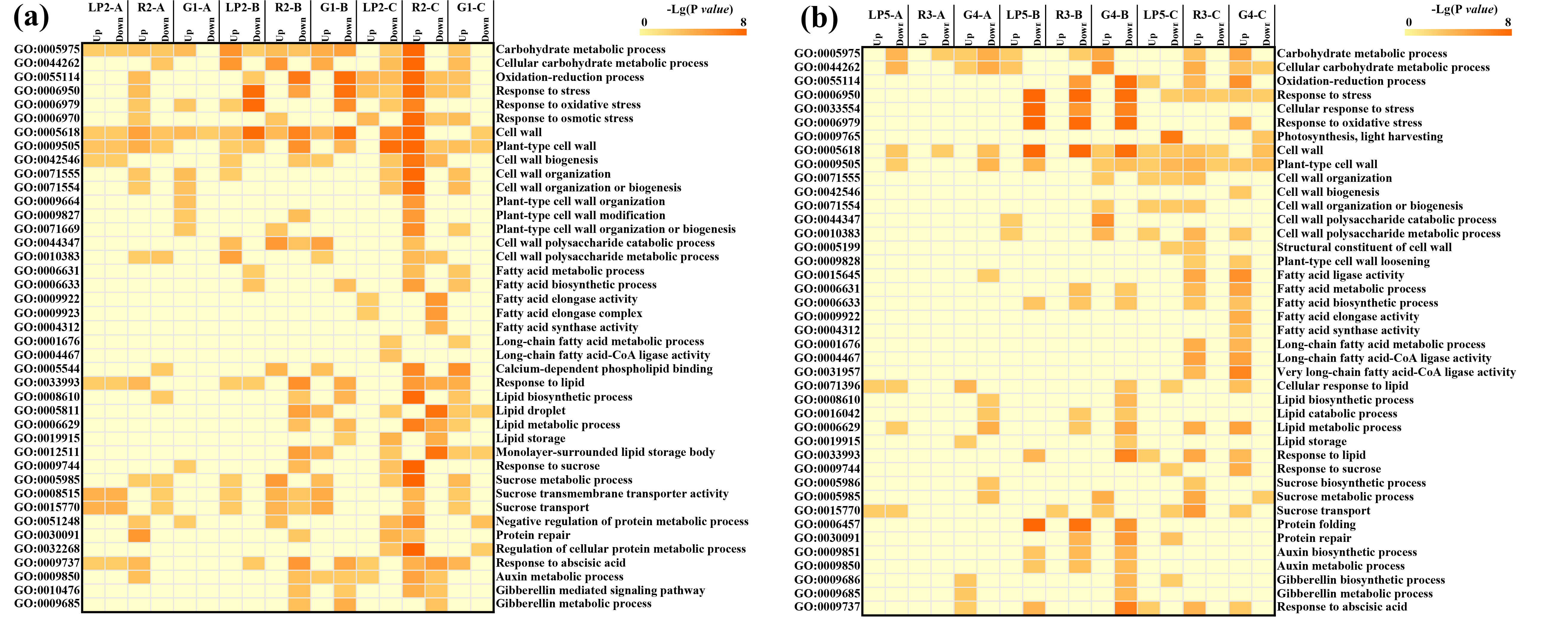
**

**Figure S15.** Enriched GO terms at different stages of seed development in *BnLPAT2* and *BnLPAT5* transgenic lines. (a) and (b) represent the enriched GO terms at different stages of seed development in down- and up-regulated genes in *BnLPAT2* and *BnLPAT5* transgenic lines, respectively. The color bars at the upper right in (a) and (b) represent the significance (corrected P-value).

**

**

**Figure S16.** Differential expression of the representative genes involved in different pathways during seed development. Expression profiles of genes involved in lipid metabolism, secondary metabolism, protein metabolism and hormone metabolism during different stages of seed development in all *BnLPAT2* and/or *BnLPAT5* lines have been shown.


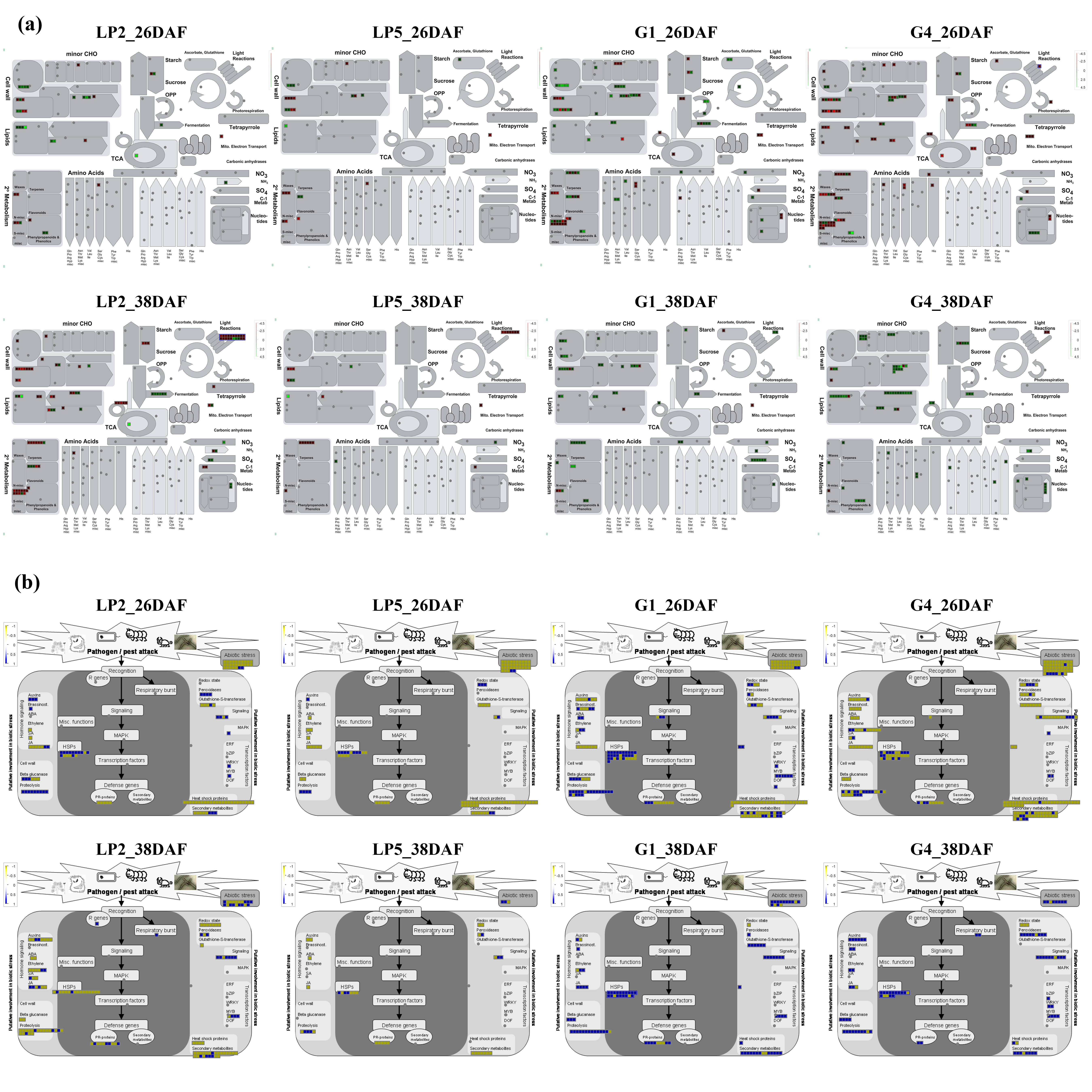


**Figure S17.** Metabolic pathways and biotic stress pathways showing differential expression in *BnLPAT2* and *BnLPAT5* lines at 26 DAF and 38DAF. (a) and (b) represent the detailed metabolic pathways and biotic stress pathways, respectively. DEGs (fold change ≥2, q-value ≤0.05) in *BnLPAT2* and *BnLPAT5* transgenic lines at 26 DAF and 38 DAF were loaded into MapMan to generate the overview. On the log2 scale in metabolic pathways, green and red colors represent higher and lower expression compared to WT, respectively. The log2 scale in biotic stress pathways, blue and yellow colors represent higher and lower expression compared to WT, respectively.
